# Apolipoprotein E is an HIV-1-inducible inhibitor of viral production and infectivity in macrophages

**DOI:** 10.1371/journal.ppat.1007372

**Published:** 2018-11-29

**Authors:** Rokeya Siddiqui, Shinya Suzu, Mikinori Ueno, Hesham Nasser, Ryota Koba, Farzana Bhuyan, Osamu Noyori, Sofiane Hamidi, Guojun Sheng, Mariko Yasuda-Inoue, Takayuki Hishiki, Sayaka Sukegawa, Eri Miyagi, Klaus Strebel, Shuzo Matsushita, Kunitada Shimotohno, Yasuo Ariumi

**Affiliations:** 1 Center for AIDS Research, Kumamoto University, Kumamoto, Japan; 2 International Research Center for Medical Sciences (IRCMS), Kumamoto University, Kumamoto, Japan; 3 Department of Clinical Pathology, Faculty of Medicine, Suez Canal University, Ismailia, Egypt; 4 Laboratory of Veterinary Microbiology, Department of Veterinary Medicine, College of Bioresource Sciences, Nihon University, Fujisawa, Kanagawa, Japan; 5 Department of Microbiology and Cell Biology, Tokyo Metropolitan Institute of Medical Science, Tokyo, Japan; 6 National Institute of Allergy and Infectious Diseases, National Institutes of Health, Bethesda, Maryland, United States of America; 7 Research Center for Hepatitis and Immunology, National Center for Global Health and Medicine, Chiba, Japan; Loyola University Chicago, UNITED STATES

## Abstract

Apolipoprotein E (ApoE) belongs to a class of cellular proteins involved in lipid metabolism. ApoE is a polymorphic protein produced primarily in macrophages and astrocytes. Different isoforms of ApoE have been associated with susceptibility to various diseases including Alzheimer’s and cardiovascular diseases. ApoE expression has also been found to affect susceptibility to several viral diseases, including Hepatitis C and E, but its effect on the life cycle of HIV-1 remains obscure. In this study, we initially found that HIV-1 infection selectively up-regulated ApoE in human monocyte-derived macrophages (MDMs). Interestingly, ApoE knockdown in MDMs enhanced the production and infectivity of HIV-1, and was associated with increased localization of viral envelope (Env) proteins to the cell surface. Consistent with this, ApoE over-expression in 293T cells suppressed Env expression and viral infectivity, which was also observed with HIV-2 Env, but not with VSV-G Env. Mechanistic studies revealed that the C-terminal region of ApoE was required for its inhibitory effect on HIV-1 Env expression. Moreover, we found that ApoE and Env co-localized in the cells, and ApoE associated with gp160, the precursor form of Env, and that the suppression of Env expression by ApoE was cancelled by the treatment with lysosomal inhibitors. Overall, our study revealed that ApoE is an HIV-1-inducible inhibitor of viral production and infectivity in macrophages that exerts its anti-HIV-1 activity through association with gp160 Env via the C-terminal region, which results in subsequent degradation of gp160 Env in the lysosomes.

## Author summary

Apolipoprotein E (ApoE) has been shown to play an important role in lipid metabolism, progression of cardiovascular and Alzheimer’s disease, and the pathogenesis of several infectious diseases such as HIV-1. However, how ApoE affects HIV-1 replication remained obscure. Here, we show that ApoE is an HIV-1-inducible inhibitor of HIV-1 production and infectivity in macrophages, one of major HIV-1 target cells. Mechanistically, ApoE appears to reduce viral infectivity by inducing lysosomal degradation of the HIV-1 envelope (Env) glycoprotein, which is essential for the entry of HIV-1 into target cells, through the intracellular association of ApoE with HIV-1 Env. Unlike the lytic infection of CD4^+^ T cells, HIV-1-infected macrophages are generally resistant to viral-induced cytopathic effects and thereby persist in tissue for extended periods of time with large numbers of infectious particles contained within cytoplasmic vacuoles. Therefore, our finding that HIV-1 up-regulates ApoE expression, which in turn limits viral spread by inhibiting Env expression, may help understand the molecular basis by which macrophages can maintain the long-term persistent infection of HIV-1.

## Introduction

Apolipoprotein E (ApoE) is involved in several biological functions, including lipid metabolism, cardiovascular diseases, Alzheimer’s disease, immune regulation, and infectious diseases [1–5]. ApoE is a component of very low density lipoprotein (VLDL), chylomicron, intermediate density lipoprotein (IDL), and high density lipoprotein (HDL) [6] and mediates the transport and uptake of cholesterol and triglycerides [1, 2]. Originally referred to as the arginine-rich apoprotein [7], ApoE is a secreted 34 kDa protein of 299 amino acid residues derived from a 317 amino acid precursor protein that is cleaved to release an 18 amino acids N-terminal signal peptide [8, 9]. The human *ApoE* gene is located on chromosome 19 in a cluster with *ApoCI* and *ApoCII* genes [10, 11]. ApoE is produced by liver, kidney, brain, and macrophages, and has three isoforms, ApoE2, ApoE3, and ApoE4, with isoform-specific functional properties [12–16]. ApoE3 and ApoE4 bind to the LDL receptor with similar affinity as a ligand whereas ApoE2 does not bind to the LDL receptor [17]. The frequencies of these alleles vary in humans (E2, 5–10%; E3, 65–80%; and E4, 13–20%), and ApoE4 allele is more frequent in African people, for instance [1–5]. Yet, they differ only by two amino acids at residues 112 and 158. ApoE2 has cysteine and ApoE4 has arginine at both positions, and ApoE3 has cysteine 112 and arginine 158 [1, 2, 6, 7]. All other animals, including the great apes, have a single isoform with arginine at the corresponding positions [18]. Plasma ApoE is synthesized primarily by liver hepatocytes and accounts for ~75% of the body’s ApoE production with the remainder synthesized by the brain and various macrophages throughout the body [12–16].

Monocytes and macrophages are one of the initial target cell types of human immunodeficiency virus type 1 (HIV-1), and act both as reservoirs and sources of virus dissemination to other tissues throughout all stages of infection [19]. HIV-1-infected macrophages are resistant to virus-induced cytopathic effects. They persist as long-term reservoirs for HIV-1 and contribute to the pathogenesis of acquired immune deficiency syndrome (AIDS) during all stages of infection. While HIV infection disrupts macrophage effector functions like phagocytosis, intracellular killing, and antigen presentation [20], virus infection induces interferon (IFN) and an array of genes that promote broad anti-viral defense and innate immune response [21, 22]. In fact, IFN-α inhibits HIV-1 infection in primary monocyte-derived macrophages (MDMs) and the monocytic THP-1 cells [23]. IFN-α treatment induces IFN-stimulated genes (ISGs) that encode anti-viral proteins [22]. These include host restriction factors such as APOBEC3G [24–26], TRIM5α [27], Tetherin (also known as BST-2) [28, 29], SAMHD1 [30, 31], and MX2 [32–34], which have been reported to restrict HIV-1 infection and replication [35]. Furthermore, a number of additional host restriction factors, including APOBEC3 family [36, 37], rhesus macaque TRIM5α [38], p21 [39, 40], DCAF1 [41], TRIM22 (also known as Staf50) [42], peroxisome proliferator-activated receptor [43, 44], urokinase-type plasminogen activator [45], viperin [46], CCAAT/enhancer binding protein [47], miR-198 [48], MARCH8 [49], and mannose receptor 1 [50] have been shown to restrict HIV replication in monocytes and macrophages [21].

ApoE is involved in the pathogenesis of infectious diseases as well as susceptibility to pathogens, including herpes simplex virus-1 (HSV-1), hepatitis C virus (HCV), hepatitis E virus (HEV), HIV-1, varicella zoster virus (VZV), and Epstein-Barr virus (EBV), malaria, *Listeria monocytogenes* (LM), and *Klebsiella pneumoniae* [51–66]. ApoE4, in particular, has been shown to affect disease progression in HSV-1-, HCV-, and HIV-1-associated diseases. It is a risk factor for Alzheimer’s disease in patients with HSV-1 in the brain [51] and facilitates HSV-1 latency in the brain [52]. Similarly, HIV-1-infected patients with the ApoE4 allele have higher rates of dementia and peripheral neuropathy [53]. Conversely, ApoE4 protects against severe liver disease caused by HCV [56] and is required for infectivity and infectious viral particle production of HCV [57, 58]. Furthermore, ApoE3 and E4 are significantly associated with protection against HEV infection [59]. Interestingly, it was reported that purified recombinant ApoE4 proteins enhance the *in vitro* HIV-1 entry using SupT1-CCR5 cells [55]. On the other hand, it was also reported that ApoE-derived antimicrobial peptide analogues inhibit HSV-1 and HIV-1 entry as well as *Pseudomonas aeruginosa* and *Staphylococcus aureus* [64, 65]. The reason for this discrepancy regarding the role of ApoE in HIV-1 infection is not clear yet. More interestingly, it was reported that ApoE was detectable in HIV-1 virions derived from HIV-1-infected MDMs [66]. However, it has not been explored whether ApoE functions as a stimulator or an inhibitor of HIV-1 infection in MDMs. Therefore, in the current study, we attempted to clarify the potential role of ApoE in the HIV-1 life cycle in MDMs.

## Results

### HIV-1 infection, but not IFN-α, up-regulates the endogenous ApoE expression in human primary monocyte-derived macrophages (MDMs)

**Fig 1:**
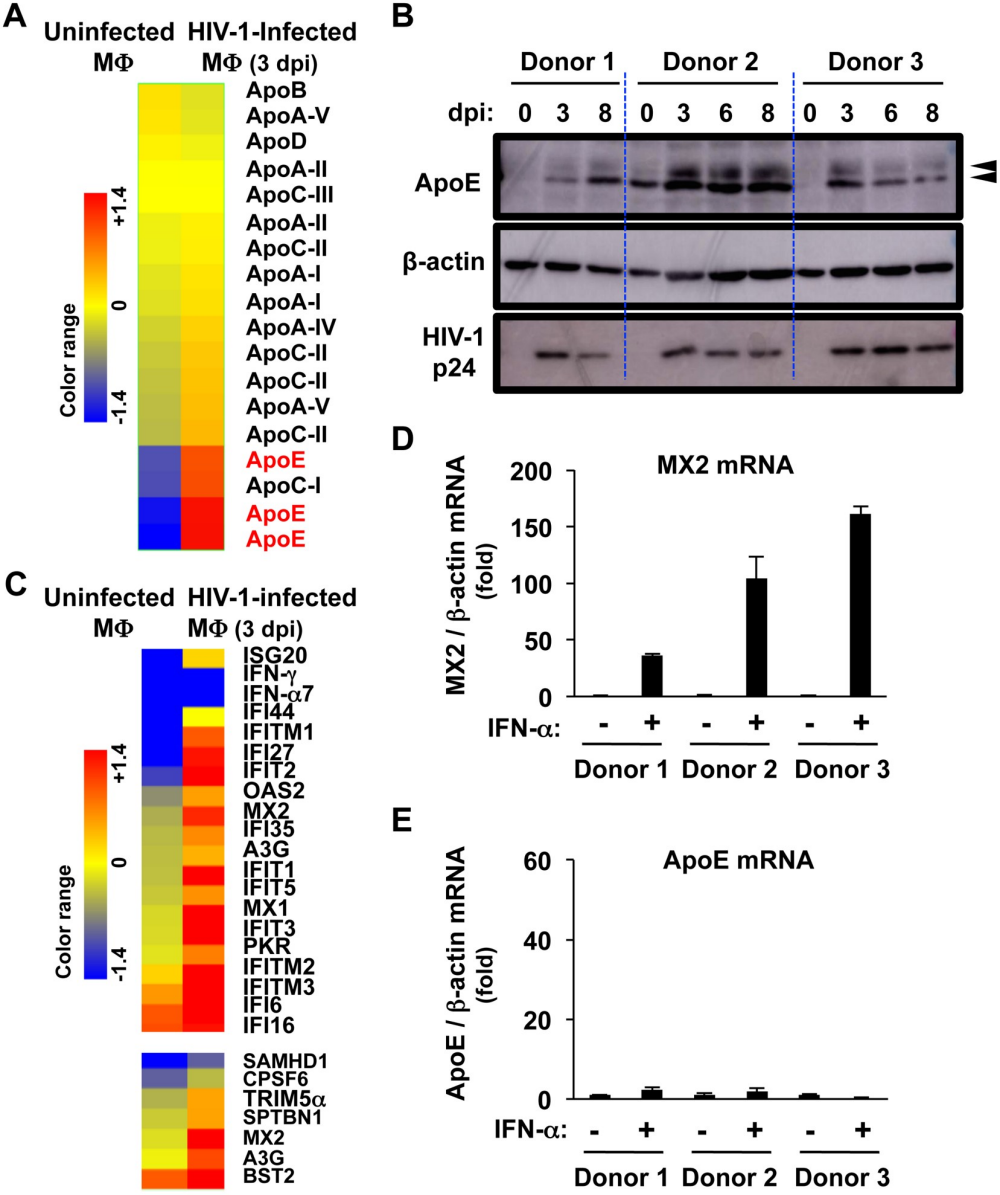
Effect of HIV-1 infection on ApoE expression in MDMs. **(A)** Heat map showing the up-regulation of *ApoE* expression (indicated in red) among *Apo* genes in HIV-1-infected MDMs were generated from the microarray analysis. The color bar indicates gene expression in the log2 scale. MDMs prepared from a healthy donor were infected with HIV-1 JR-FL (100 ng/mL p24) by using the supernatants of HEK293A cells transfected with the HIV-1 molecular clone as a source of viruses, and cultured for 3 days prior to total RNA isolation (right). The control uninfected MDMs were prepared by culturing with media for 3 days (left). dpi, days post-infection.**(B)** MDMs (3 donors) were infected with HIV-1 JR-FL as in (A), cultured for 3, 6 or 8 days, lysed, and subjected to western blot to analyze the expression of ApoE. HIV-1 p24 levels were also analyzed to verify the viral replication. Anti-β-actin blot was used as a loading control. The total cell lysates of MDMs were also prepared immediately before HIV-1 infection as a control (“0 dpi”). The arrowheads indicate the high and low molecular weight ApoE. **(C)** Heat maps showing the up-regulation of various IFN-stimulated genes (ISGs) including HIV-1 restriction factors such as *MX2*, *APOBEC3G (A3G)*, *BST-*2 and *IFITMs* were generated from the microarray analysis as in (A). **(D, E)** MDMs (3 donors) were left untreated or treated with 1,000 U/mL IFN-, cultured for 24 h, and subjected to qRT-PCR to analyze the mRNA level of *MX2* (panel D) or *ApoE* (panel E) followed by the normalization to the mRNA level of *β-actin*. Each mRNA level was calculated relative to untreated MDMs (fold). Error bars in these panels indicate standard deviation of triplicate PCR assays.

In this study, we initially investigated the effect of HIV-1 infection on endogenous *ApoE* expression in MDMs by using the JR-FL strain of HIV-1 [67]. First, the microarray analysis (Fig 1A) revealed that HIV-1 infection up-regulated the expression of *ApoE* in MDMs at 3 days post-infection (dpi). This was specific to *ApoE* because such up-regulation was not seen with other apolipoprotein genes including *ApoA-I*, *ApoA-II*, *ApoA-IV*, *ApoA-V*, *ApoB*, *ApoC-I*, *ApoC-II*, *ApoC-III* and *ApoD* (Fig 1A). This was further confirmed at the protein levels by western blot: HIV-1 infection significantly up-regulated endogenous ApoE expression in all 3 donors tested (Fig 1B). The active viral replication was confirmed by the expression of HIV-1 p24 Gag proteins (Fig 1B). In this experiment, similar numbers of MDMs (approximately 1x10^5^ cells per well in 24-well plate) were used, as evidenced by the comparable signal of β-actin among donors. Yet, the signal of ApoE in uninfected MDMs of donor 2 was higher than that of donors 1 and 3, indicating that the basal expression level of ApoE varied among donors. The multiple bands of ApoE (indicated by arrowheads) might be explained by post-translational modifications, but both high- and low molecular weight forms of ApoE were up-regulated in HIV-1-infected MDMs. The microarray analysis also showed that HIV-1 infection induced various IFN-stimulated genes (ISGs) including HIV-1 restriction factors such as *MX2*, *APOBEC3G (A3G)*, *BST2*, and *IFITMs* in MDMs (Fig 1C). Likewise, IFN-α treatment markedly enhanced *MX2* expression in MDMs (Fig 1D). In contrast, *ApoE* expression was not affected by IFN-α (Fig 1E). Thus, *ApoE* is not an ISG but an un-reported HIV-1-inducible cellular gene in MDMs.

To further confirm the ApoE induction by HIV-1, we performed several experiments. In the experiments shown in Fig 1A and 1B, the supernatants of HEK293A cells transfected with the HIV-1 molecular clones were used as a source of recombinant viruses. However, when MDMs were incubated with the supernatants of HEK293A cells transfected with the empty vector (see “Mock”), ApoE induction was never detected even when monitored up to day 8 (S1 Fig), confirming that the observed change in ApoE levels was caused by HIV-1 infection. These experiments also showed that the ApoE induction by HIV-1 was detectable as early as 1 dpi, suggesting that ApoE is an HIV-1-inducible early cellular factor. In contrast, ApoB, a primary apolipoprotein of chylomicrons, VLDL, IDL, and LDL, was not induced by HIV-1 infection (S1 Fig), since ApoB is expressed primarily in liver and small intestine, but not in other tissues [68]. We also found that the ApoE induction was independent of HIV-1 accessary proteins including Nef, Vpr, Vpu and Vif, because the mutant viruses lacking the expression of each viral gene still retained the ability to up-regulate ApoE (S2 Fig). Furthermore, we found that the ApoE was never induced in either the T cell line (MT-4 cells) or primary peripheral CD4^+^ T cells, in spite of active viral replication as evidenced by p24 expression in these cells (S3 Fig). Thus, these results indicate that HIV-1 replication itself up-regulates ApoE expression in macrophages but not in T lymphocytes, which is independent of HIV-1 proteins including Nef, Vpr, Vpu and Vif.

### ApoE knockdown in MDMs results in an enhancement of HIV-1 production

**Fig 2:**
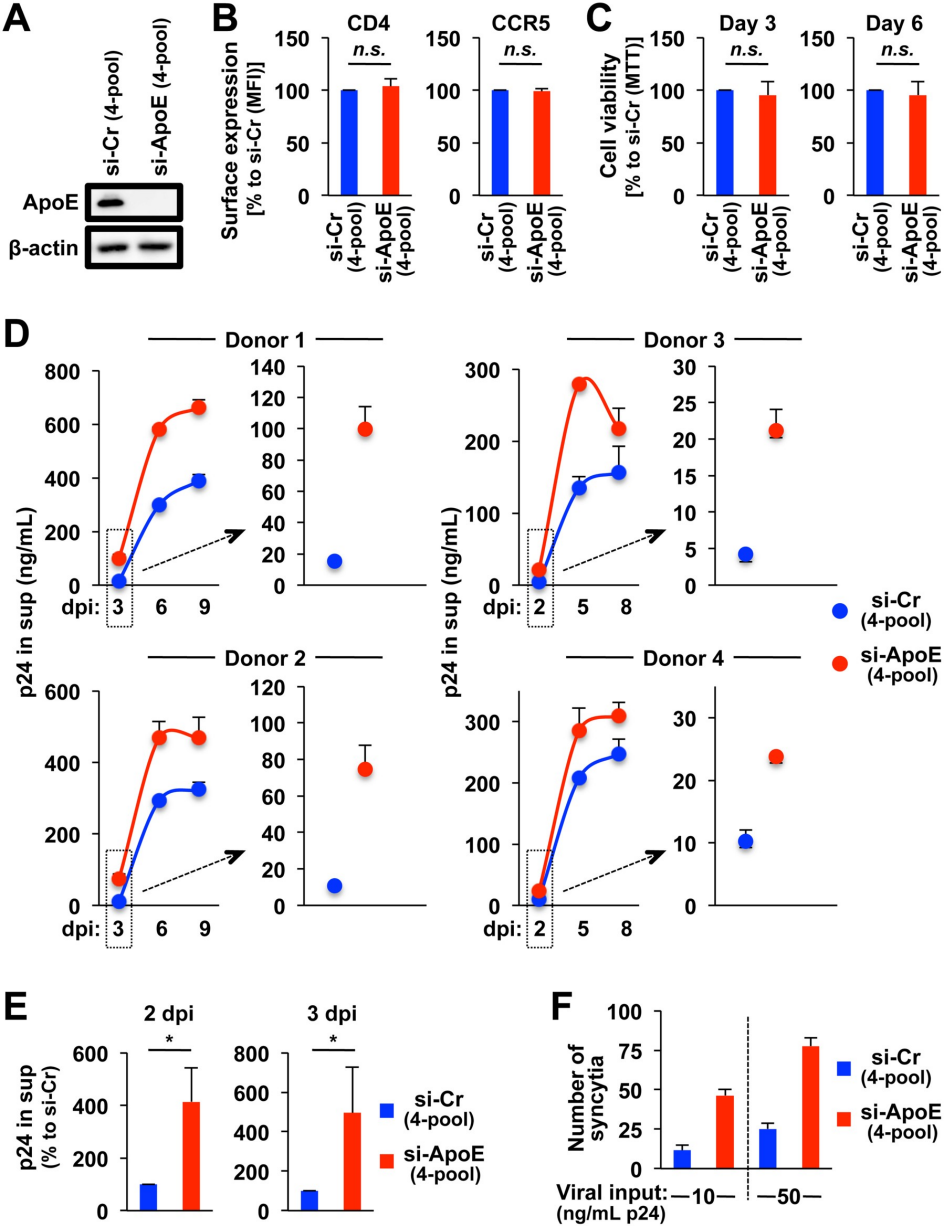
Effect of ApoE knockdown on HIV-1 production in MDMs. **(A)** MDMs were transfected with either ApoE-targeting siRNA (“si-ApoE”) or non-targeting siRNA as a control (“si-Cr”), which is a mixture (SMARTpool) of 4 siRNAs (“4-pool”), cultured for 2 days, and analyzed for their ApoE levels by western blot. Anti-β-actin blot was used as a loading control. Data shown are representative of experiments obtained from 4 different donors with similar results. **(B)** MDMs were transfected as in (A), cultured for 2 days, and analyzed for their cell surface CD4 (left panel) and CCR5 (right panel) expression by flow cytometry. The mean fluorescence intensity (MFI) values of ApoE siRNA-transfected cells are represented as percentages relative to those of control siRNA-transfected cells. Results for MDMs obtained from 3 different donors are summarized. *n*.*s*., not significant. **(C)** MDMs were transfected as in (A), cultured for 3 (left panel) or 6 days (right panel), and analyzed for their viability by the MTT assay. The viabilities of ApoE siRNA-transfected cells are represented as percentages relative to those of control siRNA-transfected cells. Results for MDMs obtained from 3 different donors are summarized. *n*.*s*., not significant. **(D)** MDMs (4 donors) were transfected as in (A), cultured for 2 days, and infected with HIV-1 JR-FL (100 ng/mL p24). The culture supernatants (“sup”) were collected as indicated (dpi, days of post-infection), and analyzed for their levels of p24 concentration by ELISA. The left panel in each donor set shows the overall kinetics of viral production. The right panel in each donor set shows the p24 concentrations in an earlier phase (3 dpi for donors 1 and 2, or 2 dpi for donors 3 and 4). **(E)** MDMs were transfected as in (A), infected as in (D), and analyzed for their levels of p24 concentration in the supernatants by ELISA. The p24 values of ApoE siRNA-transfected cells are represented as percentages relative to those of control siRNA-transfected cells. Results for MDMs obtained from 4 donors are summarized. *p < 0.05. **(F)** MDMs were transfected as in (A), cultured for 2 days, infected with HIV-1 JR-FL with different viral inputs (10 or 50 ng/mL of p24) for 2 days, and stained with DAPI. The numbers of fused MDMs (“syncytia”) that had more than 5 nuclei were quantified by selecting 3 different areas for each group (see S4 Fig for typical multi-nucleated MDMs). Data shown are representative of experiments obtained from 2 different donors with similar results.

The selective ApoE induction in MDMs by HIV-1 at both mRNA and protein levels prompted us to investigate the role of ApoE in HIV-1 replication in the cells. To this end, we employed the transient knockdown of endogenous ApoE using siRNA. Since the basal expression level of ApoE varied among donors (see Fig 1B), we performed the knockdown experiments using donors whose basal ApoE levels were relatively high, and confirmed an effective knockdown of ApoE in MDMs (Fig 2A). In this experiment, we used ApoE-targeting siRNA (“si-ApoE”) and non-targeting siRNA as a control (“si-Cr”), which is a mixture (SMARTpool) of 4 siRNAs (“4-pool”). The ApoE knockdown affected neither the cell surface expression of HIV-1 receptors such as CD4 and CCR5 (Fig 2B) nor the viability of MDMs (Fig 2C). Interestingly, the levels of p24 in the supernatants (“sup”) of MDMs transfected with ApoE siRNA were higher than those of MDMs transfected with control siRNA in all 4 donors tested (Fig 2D), which was more evident at 2 or 3 dpi (the right panel in each donor set of Fig 2D and 2E) than 5 or 6 dpi (the left panel in each donor set of Fig 2D). In fact, when assessed at 2 dpi, the cultures of MDMs transfected with ApoE siRNA contained higher number of multi-nucleated fused MDMs (the formation of “syncytia”), which is a hallmark of an active viral replication [69], than those of MDMs transfected with control siRNA (Fig 2F and S4 Fig). Thus, these results suggest that ApoE has an anti-HIV-1 activity, in particular, in earlier infection phases such as 2 and 3 dpi, which was consistent with the finding that the ApoE induction by HIV-1 was detectable as early as 1 dpi (see S1 Fig).

**Fig 3:**
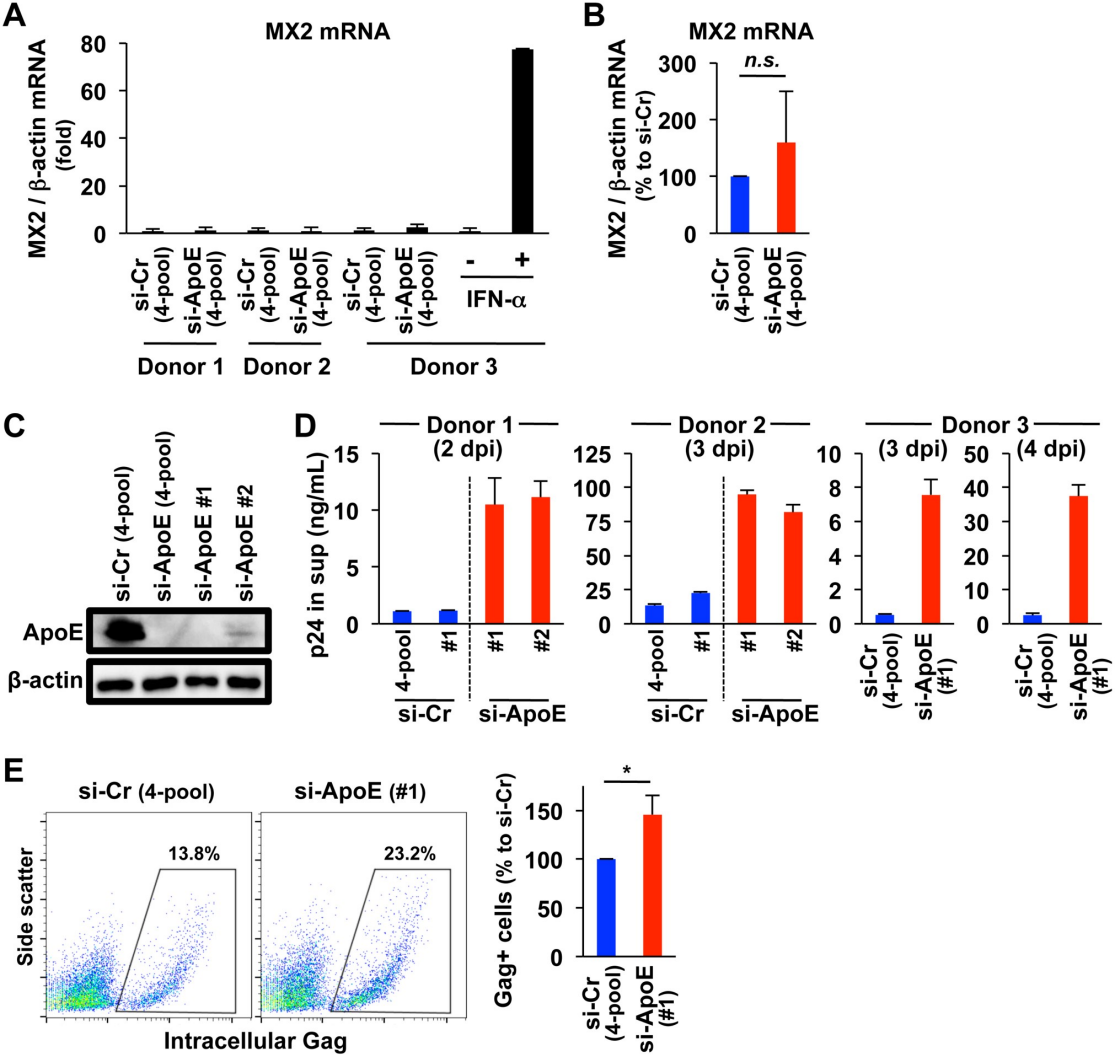
Effects of ApoE knockdown on MX2 expression and HIV-1 production in MDMs. **(A)** MDMs (3 donors) were transfected with either ApoE-targeting siRNA (“si-ApoE”) or non-targeting siRNA as a control (“si-Cr”), which is a mixture (SMARTpool) of 4 siRNAs (“4-pool”), cultured for 2 days, and analyzed for their *MX2* mRNA levels by qRT-PCR followed by the normalization to the mRNA level of *β-actin*. Each mRNA level was calculated relative to the control siRNA-transfected MDMs (fold). Error bars in these panels indicate standard deviation of triplicate PCR assays. MDMs left untreated or treated with IFN-α for 24 h were added as a reference for *MX2* expression (see donor 3). **(B)** MDMs were transfected and analyzed as in (A). The *MX2* mRNA expression levels of ApoE siRNA-transfected cells are represented as percentages relative to those of control siRNA-transfected cells. Results for MDMs obtained from 3 different donors are summarized. *n*.*s*., not significant. **(C)** MDMs were transfected with either ApoE-targeting siRNA (“si-ApoE”) or non-targeting siRNA as a control (“si-Cr”), cultured for 2 days, and analyzed for their ApoE levels by western blot. In addition to the pool of 4 (#1, #2, #3 and #4) ApoE-targeting siRNAs (“4-pool”), #1 and #2 siRNAs were used. Anti-β-actin blot was used as a loading control. Data shown are representative of experiments obtained from 4 different donors with similar results. **(D)** MDMs (3 donors) were transfected with the indicated siRNAs (ApoE-targeting or non-targeting siRNA), cultured for 2 days, and infected with HIV-1 JR-FL (100 ng/mL p24). The culture supernatants were collected as indicated, and analyzed for their levels of p24 concentration by ELISA. **(E)** MDMs were transfected with either ApoE-targeting siRNA (#1) or non-targeting siRNAs (SMART pool), cultured for 2 days, and infected with HIV-1 JR-FL (100 ng/mL p24) for another 2 days. Then, their intracellular Gag levels were determined by flow cytometry. In the right panel, the frequencies of intracellular Gag-positive MDMs in the ApoE siRNA transfection are represented as percentages relative to those in the control siRNA transfection, and results for MDMs obtained from 3 donors are summarized. *p < 0.05.

To further confirm the anti-HIV-1 activity of ApoE, we performed several experiments. First, we compared the levels of *MX2*, the typical ISG (see Fig 1D), between MDMs transfected with ApoE siRNA and those transfected with control siRNA, using qRT-PCR. As shown (Fig 3A and 3B), we did not find any statistical difference in *MX2* expression between 2 groups: at least, the level of *MX2* mRNA in MDMs transfected with ApoE siRNA was not higher than that of MDMs transfected with control siRNA. Thus, it was likely that the enhanced HIV-1 production observed by ApoE knockdown was not due to an induction of ISGs. Next, we attempted to confirm the enhanced viral production by ApoE knockdown using additional siRNAs. Among the pool of 4 siRNAs (#1, #2, #3 and #4) that was used in the experiments shown above, #1 and #2 siRNAs were sufficient for effective knockdown of ApoE (Fig 3C). As shown (Fig 3D), the levels of p24 in the supernatants of MDMs transfected with ApoE #1 siRNA or #2 siRNA were still higher than those of MDMs transfected with control non-targeting siRNA (the pool or #1). Thus, it was likely that the enhanced HIV-1 production observed by ApoE knockdown was not due to an off-target effect of the siRNAs used. Interestingly, in addition to p24 Gag in the supernatants, the levels of intracellular Gag in MDMs transfected with ApoE siRNA was also higher than those in MDMs transfected with control non-targeting siRNA (Fig 3E), indicating that ApoE knockdown indeed enhanced viral production, but not simply due to an enhancement of viral release.

### ApoE knockdown in MDMs also results in an enhancement of the infectivity of produced viruses and the cell surface expression of HIV-1 envelope (Env) proteins

**Fig 4:**
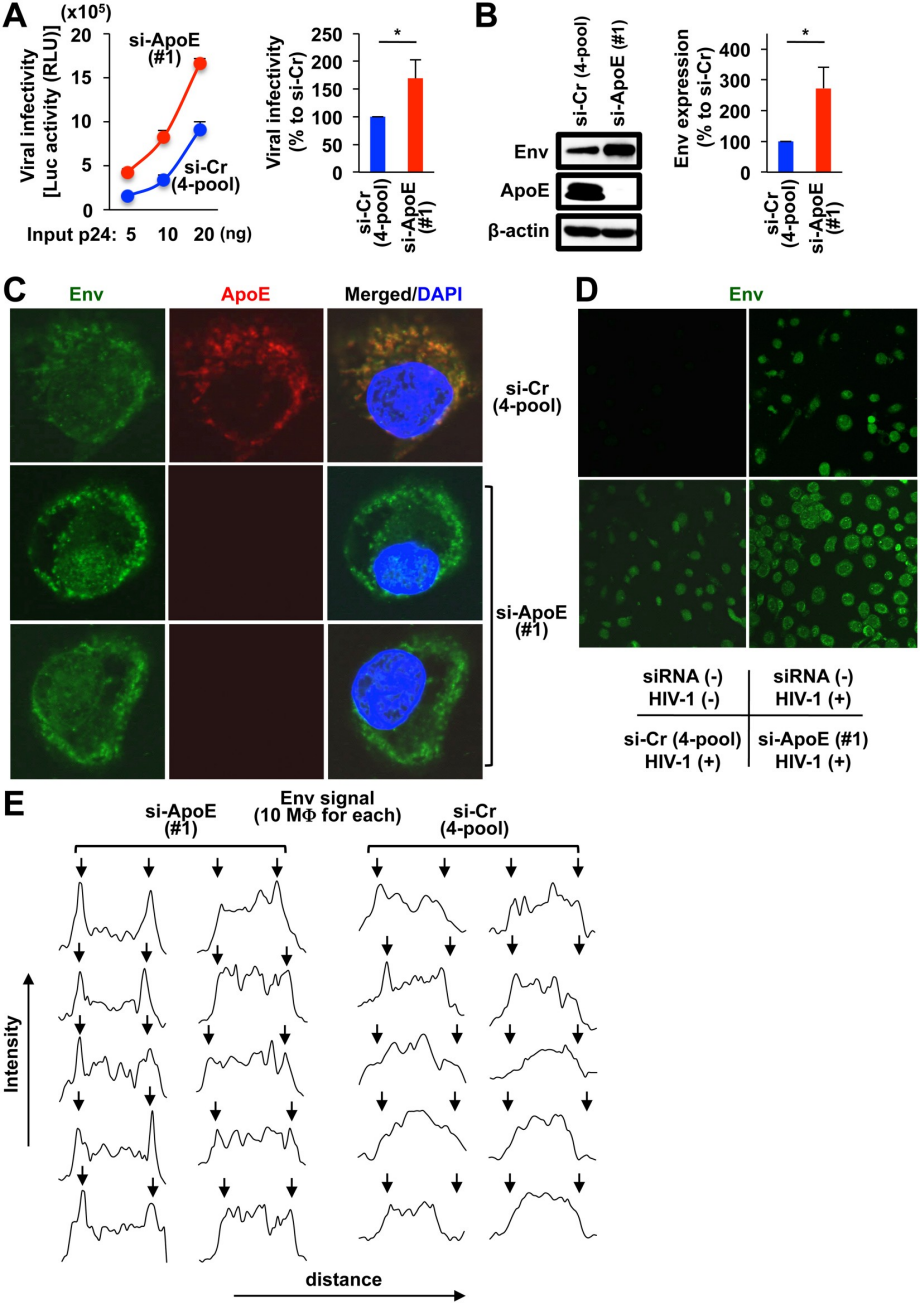
Effects of ApoE knockdown on infectivity of produced HIV-1 and localization of HIV-1 Env in MDMs. **(A)** MDMs were transfected with either ApoE siRNA #1 or pooled non-targeting siRNAs, cultured for 2 days, and infected with HIV-1 JR-FL (100 ng/mL p24). In the left panel, the supernatants were collected at 2 dpi, and the infectivity of the produced viruses was analyzed by the luciferase (Luc) reporter gene assay with TZM-bl cells, by changing the viral input (p24 amount) as indicated. RLU, relative light units. In the right panel, the infectivity of the viruses produced by ApoE siRNA-transfected cells are represented as percentages relative to that by control siRNA-transfected cells, and results for MDMs obtained from 3 donors are summarized. *p < 0.05. **(B)** MDMs were transfected and infected as in (A). In the left panel, the cell lysates were prepared at 2 dpi, and their levels of Env (gp160) were analyzed by western blot. ApoE levels were also analyzed to verify the knockdown efficiency, and anti-β-actin blot was used as a loading control. In the right panel, the Env levels (quantified by the densitometric analysis) in ApoE siRNA-transfected cells are represented as percentages relative to those in control siRNA-transfected cells, and results for MDMs obtained from 3 donors are summarized. *p < 0.05. **(C)** MDMs were transfected with either pooled non-targeting siRNAs (top panels) or ApoE siRNA #1 (middle and bottom panels), cultured for 2 days, infected with HIV-1 JR-FL (100 ng/mL p24) and co-stained with anti-Env antibodies (green), anti-ApoE antibodies (red) and DAPI (blue) at 2 dpi. Data shown are representative of experiments obtained from 2 different donors with similar results. Original magnification x600. **(D)** MDMs were stained with anti-Env antibodies (green). MDMs were prepared as follows: left untransfected and uninfected (upper left), untransfected and infected with HIV-1 JR-FL (100 ng/mL p24) (upper right), transfected with pooled non-targeting siRNAs and infected with HIV-1 JR-FL (100 ng/mL p24) (lower left), and transfected with ApoE siRNA #1 and infected with HIV-1 JR-FL (100 ng/mL p24) (lower right). MDMs were cultured for 2 days after the transfection, and then analyzed for Env expression at 2 dpi. Data shown are representative of experiments obtained from 2 different donors with similar results. **(E)** MDMs were transfected with either ApoE siRNA #1 (left) or pooled non-targeting siRNAs (right), and infected and stained as in (D). MDMs were randomly selected (10 cells for each group) and analyzed for their distribution of Env signal. The position of the cell surface are indicated by arrows.

Interestingly, we also found that the infectivity of the viruses produced by ApoE siRNA-transfected MDMs was higher than that by control siRNA-transfected MDMs (Fig 4A, left graph). The difference in the infectivity between 2 groups was relatively small when compared with that in the viral production (see Figs 2E and 3D), but statistically significant (Fig 4A, right graph). Since HIV-1 envelope (Env) proteins, which interact with HIV-1 receptors, are critical for the infectivity of virions, we hypothesized that the ApoE knockdown exerted some influence on Env. First, the levels of Env in MDMs transfected with ApoE siRNA were higher than those in MDMs transfected with control non-targeting siRNAs (Fig 4B). This was as expected, because the ApoE knockdown enhanced the viral production itself. However, of importance, we found that Env tended to localize at the surface of MDMs transfected with ApoE siRNA (Fig 4C and 4D for higher and lower magnification, respectively). The Env signal was specific because it was not detected in uninfected MDMs (Fig 4D, upper left). In the quantitative analysis (Fig 4E), approximately half of MDMs in the ApoE siRNA-transfected cultures (“si-ApoE”) showed a bright signal of Env at the cell surface (left-most set), which was contrast to the finding that most MDMs in the control cultures (“si-Cr”) showed a diffuse signal of Env. The distribution of the bright Env signal in the ApoE siRNA-transfected MDMs was similar to that of CD14, the typical cell surface marker for MDMs (S5 Fig). Thus, it was likely that the ApoE knockdown facilitated the localization of Env at the surface of MDMs, which resulted in the enhanced viral infectivity and viral replication.

### Exogenous expression of ApoE in 293T cells reduces HIV-1 Env expression and viral infectivity

**Fig 5:**
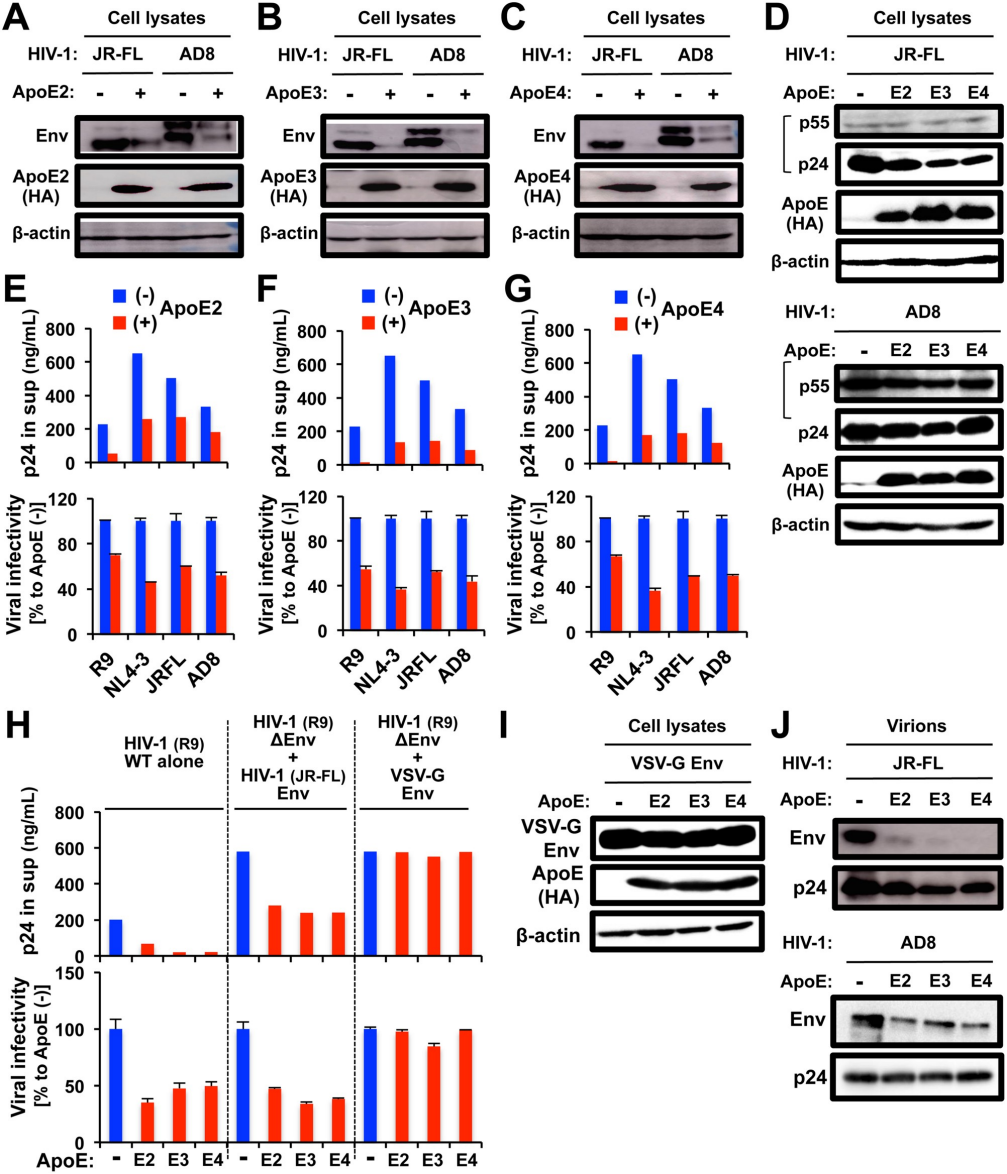
Effects of exogenous expression of ApoE on Env expression and viral infectivity in 293T cells. **(A-C)** The 293T cells were transfected with 2 μg of the HIV-1 molecular clone (JR-FL or AD8), or co-transfected with 2 μg of the indicated HA-tagged ApoE expression vector, cultured for 3 days, lysed, and subjected to western blot to analyze the expression of Env. ApoE expression was verified using anti-HA antibody. Anti-β-actin blot was used as a loading control. **(D)** The 293T cells were transfected with 2 μg of the HIV-1 molecular clone (JR-FL in upper panel, and AD8 in lower panel), or co-transfected with 2 μg of the indicated HA-tagged ApoE expression vector, cultured for 3 days, lysed, and subjected to western blot to analyze the expression of Gag (p55 and p24). **(E-G)** The 293T cells were transfected with 2 μg of the HIV-1 molecular clone (R9, NL4-3, JR-FL or AD8), or co-transfected with 2 μg of the indicated HA-tagged ApoE expression vector. The supernatants of the transfected 293T cells were collected and analyzed for their levels of p24 concentration by ELISA (upper panels). Then, viruses (input p24: 10 ng/mL) were added to TZM-bl indicator cells. At 24 h of post-infection, the viral infectivity was measured by luciferase assays (lower panels). The infectivity of the viruses produced by ApoE co-transfected cells are represented as percentages relative to that by empty vector-transfected cells. Error bars indicate standard deviations of triplicate assays. Data shown are representative of 3 independent experiments with similar results. **(H)** The 293T cells were transfected with 1 μg of the wild-type (WT) HIV-1 molecular clone (R9), 1 μg of its mutant lacking Env (R9ΔEnv) supplemented with 1 μg of HIV-1 Env expression vector (JR-FL Env) or 0.5 μg of R9ΔEnv supplemented with 0.5 μg of VSV-G Env expression vector, or co-transfected with 1 μg of the indicated HA-tagged ApoE expression vector. The supernatants of the transfected 293T cells were collected and analyzed for their levels of p24 concentration by ELISA (upper panel). Then, the infectivity of the produced viruses (lower panel) was measured as in (E-G). **(I)** The 293T cells were transfected with 2 μg of VSV-G Env expression vector, or co-transfected with 2 μg of the indicated HA-tagged ApoE expression vector, cultured for 3 days, lysed, and subjected to western blot to analyze the expression of VSV-G. **(J)** The 293T cells were transfected with 2 μg of the HIV-1 molecular clone (JR-FL in upper panel, and AD8 in lower panel), or co-transfected with 2 μg of the indicated HA-tagged ApoE expression vector, and cultured for 3 days. The culture supernatants were filtrated with a 0.45 μm filter and centrifuge at 20,000 x g for 2 h at 4 °C. The pellets were lysed and subjected to western blot to analyze the expression of HIV-1 Env and p24.

The results with MDMs suggested that ApoE, which is the newly-identified HIV-1-inducible cellular protein functions as the anti-HIV-1 factor by targeting Env. To test this hypothesis, we performed a series of ApoE over-expression studies using 293T cells, which are negative for ApoE expression (S6 Fig). We initially found that all the ApoE isoforms (ApoE2 in Fig 5A, ApoE3 in Fig 5B, and ApoE4 in Fig 5C) significantly reduced the intracellular expression of Env when co-expressed with CCR5-trpoic HIV-1 molecular clone such as JR-FL [67] and AD8 [70]. The level of exogenous ApoE expression in 293T cells was similar to that of JR-FL-infected MDMs, and the ApoE reduced the Env expression in 293T cells in a dose dependent manner (S7 Fig). In contrast, ApoE isoforms had little or modest effect on the intracellular expression of p24 and its precursor form p55 (S7 Fig and Fig 5). Of importance, all the ApoE isoforms (ApoE2 in Fig 5E, ApoE3 in Fig 5F, and ApoE4 in Fig 5G) significantly reduced not only the viral production (upper panels) but also the infectivity of produced viruses (lower panels) when co-expressed with the CCR5-trpoic HIV-1 molecular clones (JR-FL and AD8). Such reduction in the viral infectivity was also observed with CXCR4-tropic clones such as R9 and NL4-3 [71–73] (Fig 5E–5G), and the ROD10 strain [74] of HIV-2 (S8 Fig). Meanwhile, none of ApoE isoforms affected the infectivity of VSV-G Env-pseudotyped viruses (Fig 5H, right-most set) and the intracellular expression of VSV-G Env (Fig 5I), indicating that the inhibitory activity of ApoE on the expression of HIV-1 Env and its infectivity was not due to a non-specific effect. Indeed, we found that all the ApoE isoforms markedly reduced the amount of HIV-1 Env, but not p24, incorporated into virions (Fig 5J), which was highly likely to explain the low viral infectivity by ApoE co-expression. Thus, the results of both ApoE knockdown in MDMs and ApoE expression in 293T cells support the conclusion that ApoE affects Env expression and thereby infectivity of HIV-1.

### ApoE co-localizes with HIV-1 Env in the cytoplasm and associates with the gp160 Env precursor

We next attempted to clarify the molecular mechanisms by which ApoE affected the expression HIV-1 Env. First, the reduction of HIV-1 Env expression by ApoE in 293T cells was reproducible even when we used the plasmid expressing Env alone (S9A Fig). Such obvious reduction was not observed with the plasmid expressing p24 alone (S9B Fig), as expected. Meanwhile, the reduction of HIV-2 (ROD10) Env expression by ApoE in 293T cells was also reproducible even when we used the plasmid expressing Env alone (S9C Fig). Thus, ApoE affected HIV-1 and HIV-2 Env expression, even in the absence of other viral proteins.

**Fig 6:**
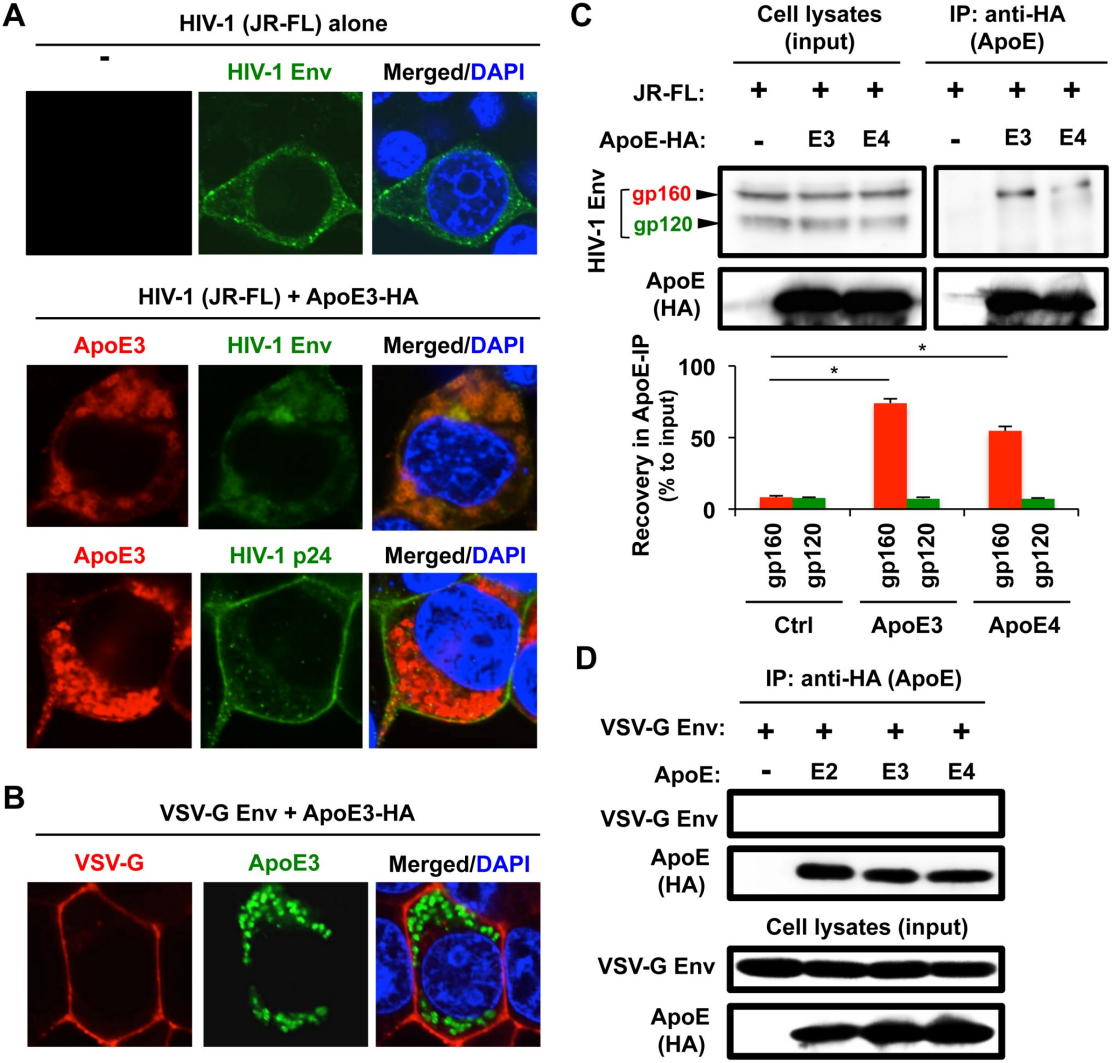
Effect of exogenous expression of ApoE on the localization of Env, and the association between ApoE and gp160 Env in 293T cells. **(A)** The 293T cells were transfected with 100 ng of the HIV-1 molecular clone JR-FL (top panels), or co-transfected with 100 ng of the HA-tagged ApoE3 expression vector (middle and bottom panels) in a 2-well chamber slide. Twenty-four hours post-transfection, the cells were stained with anti-HIV-1 Env, anti-HIV-1 p24, or anti-HA antibody, and the signals were visualized with AlexaFluor488 goat anti-Human IgG or AlexaFluor594 donkey anti-mouse IgG. Nuclei were stained with DAPI (blue). Original magnification x600. **(B)** The 293T cells were co-transfected with 100 ng of VSV-G Env expression vector and 100 ng of HA-tagged ApoE3 expression vector, in a 2-well chamber slide. Twenty-four hours post-transfection, the cells were co-stained with anti-VSV-G antibody (red), anti-HA antibody (green) and DAPI (blue). Original magnification x600. **(C)** The 293T cells were transfected with 4 μg of the HIV-1 molecular clone JR-FL or 4 μg of the indicated HA-tagged ApoE expression vector (either ApoE3 or ApoE4). The cell lysates were mixed and incubated with the anti-HA antibody (to precipitate the ApoE complex), and the ApoE immunoprecipitates (IP) were analyzed for the presence of Env by western blot (upper right). The level of Env in the total cell lysates is also shown (upper left, “input”). The arrowheads indicate the precursor (gp160) and mature form (gp120) of Env. In the bar graph, the gp160 or gp120 levels (quantified by the densitometric analysis) in the ApoE immunoprecipitates are represented as percentages relative to those in total cell lysates (”Recovery in ApoE-IP”), and results obtained from three independent experiments are summarized. *p<0.0001.**(D)** The 293T cells were transfected with 2 μg of VSV-G Env expression vector, or co-transfected with 2 μg of the indicated HA-tagged ApoE expression vector. Then, the cell lysates were immunoprecipitated as in (C), and the ApoE immunoprecipitates (IP) were analyzed for the presence of VSV-G Env by western blot (upper). The level of VSV-G Env in the total cell lysates is also shown (lower, “input”).

As mentioned above, it appeared that ApoE co-localized with HIV-1 Env in MDMs (see Fig 4C, top panels) and ApoE knockdown facilitated the localization of Env at the surface of MDMs (see Fig 4E and S5 Fig). It was shown that Env was quickly endocytosed [75]. In fact, Env localized diffusely in the cytoplasm when expressed in 293T cells (S10 Fig). However, in approximately half of the transfected 293T cells, Env localized also to the plasma membrane (see S10 Fig, left-most set, and Fig 6A, top panel). Interestingly, when co-expressed with ApoE, Env tended to localize into cytoplasmic vesicles together with ApoE, in most of the transfected 293T cells (Fig 6A, middle panels, and S10 Fig, “ApoE (+)”). Such change from the plasma membrane to cytoplasmic vesicles in the presence of ApoE was not observed with p24 (Fig 6A, bottom panels). The change in the localization of HIV-1 Env by ApoE co-expression was not due to a non-specific effect because VSV-G Env predominantly localized to the plasma membrane even in the presence of ApoE (Fig 6B). Interestingly, when the lysates of 293T cells expressing ApoE and those expressing HIV-1 Env were mixed, incubated with anti-HA antibody to immunoprecipitate the ApoE complex, and analyzed by western blot using the anti-Env monoclonal antibody KD-247 [76], we detected glycoprotein (gp)160, a precursor form of Env, in the ApoE immunoprecipitates (Fig 6C). In the experiment, ApoE and Env were expressed in 293T cells independently and their lysates were mixed for the immunoprecipitation because ApoE significantly reduced the intracellular expression of Env when co-expressed (see Fig 5). gp160 was also detected in ApoE immunoprecipitates under the different immunoprecipitation conditions (see S13 Fig for details). In contrast, VSV-G Env was not detected in the ApoE immunoprecipitates (Fig 6D), as expected. HIV-1 gp160 Env is cleaved into the mature Env proteins (gp120 and gp41) by the host cell protease furin [77], and gp120 was minimally detected in the ApoE immunoprecipitates (Fig 6C). Thus, it was likely that ApoE preferentially associated with the precursor gp160 or gp160-interacted protein(s), which explained the ApoE-Env co-localization in the cytoplasm.

### The C-terminal region of ApoE is required for its inhibitory activity to HIV-1 Env, and ApoE targets HIV-1 Env for lysosomal degradation

**Fig 7:**
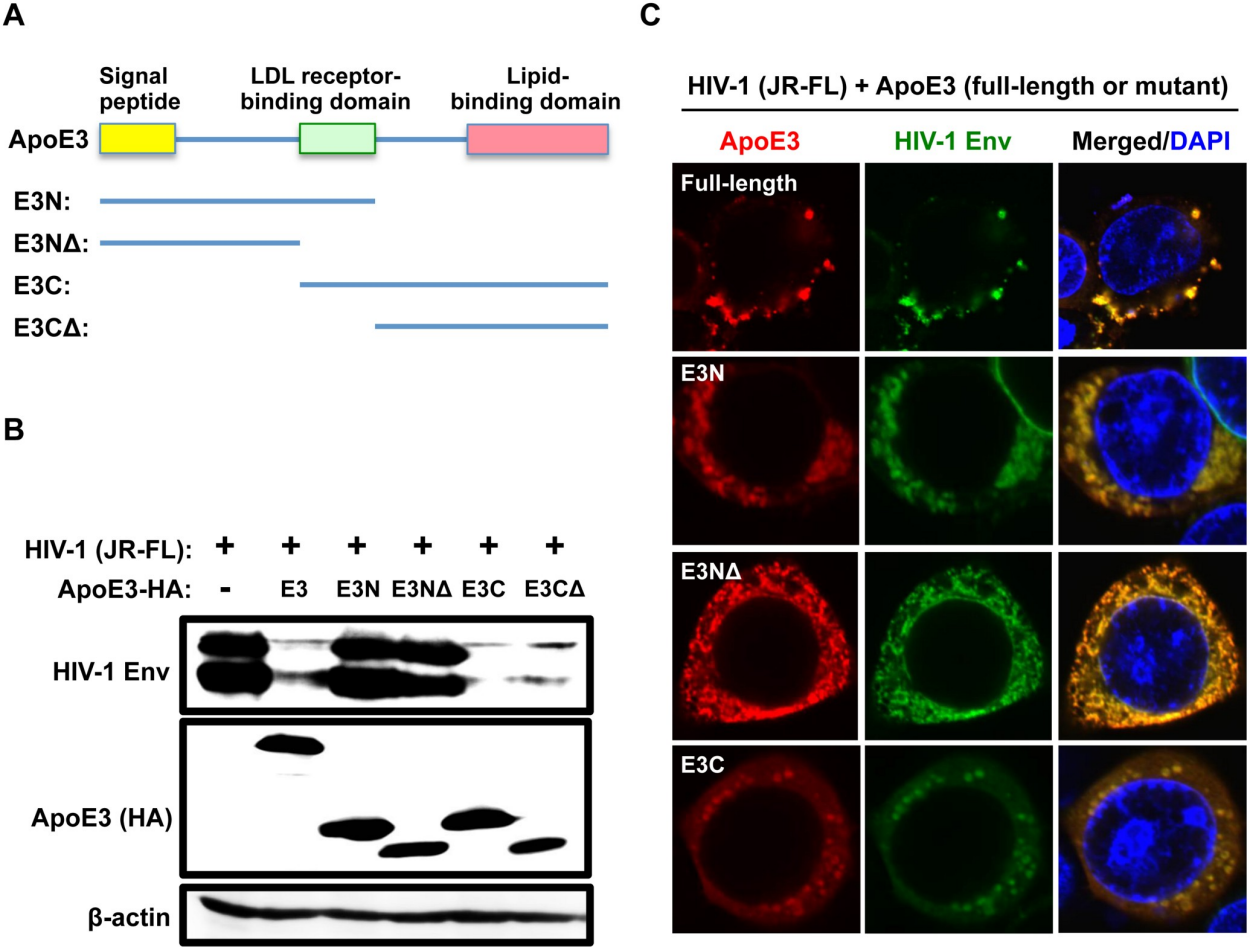
Effects of ApoE3 mutants on Env expression and localization in 293T cells. **(A)** Schematic representation of ApoE3 protein structure and the deletion mutants (E3N, E3NΔ, E3C and E3CΔ). ApoE has three functional domains: the N-terminal signaling domain, the LDL receptor-binding domain, and the C-terminal lipid-binding domain. **(B)** The 293T cells were transfected with 2 μg of the HIV-1 molecular clone JR-FL, or co-transfected with 2 μg of the HA-tagged full-length ApoE3 (E3) or the indicated mutant expression vector. The cells were then lysed, and subjected to western blot to analyze the expression of Env. ApoE expression was verified using anti-HA antibody. Anti-β-actin blot was used as a loading control. **(C)** The 293T cells were co-transfected with 100 ng of the HIV-1 molecular clone JR-FL and 100 ng of the indicated ApoE3 expression vector. Twenty-four hours post-transfection, the cells were stained with anti-HIV-1 Env and anti-HA antibodies, and the signals were visualized with AlexaFluor488 goat anti-Human IgG and AlexaFluor594 donkey anti-mouse IgG. Nuclei were stained with DAPI (blue). Original magnification x600.

**Fig 8:**
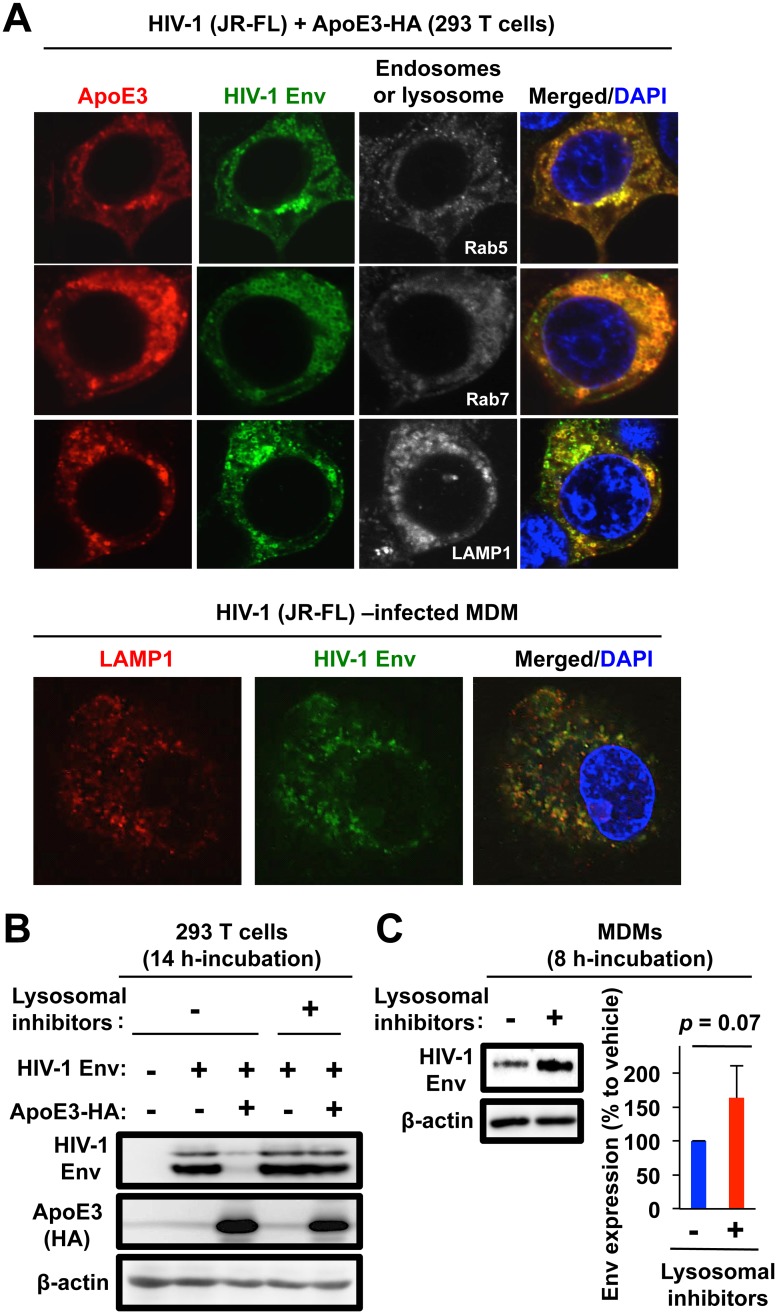
Presence of Env in endosomes and lysosomes in ApoE-expressing 293T cells and HIV-1-infected MDM, and effect of lysosomal inhibitors on ApoE-induced suppression of Env expression. **(A)** The 293T cells (upper panels) were co-transfected with 100 ng of the HIV-1 molecular clone JR-FL and 100 ng of pCAG-ApoE3-HA in a 2-well chamber slide. Twenty-four hours post-transfection, the cells were co-stained with anti-HIV-1 Env, anti-HA, and either anti-Rab5 (early endosome), anti-Rab7 (late endosome), or anti-LAMP1 (lysosome) antibodies, and then the signals were visualized with AlexaFluor488 goat anti-Human IgG, AlexaFluor594 donkey anti-mouse IgG and/or AlexaFluor647 donkey anti-rabbit IgG antibodies. MDMs (lower panels) infected with HIV-1 JR-FL (100 ng/mL p24) were also stained with anti-LAMP1 (red) and anti-Env antibodies (green) at 3 dpi. Nuclei were stained with DAPI. **(B)** The 293T cells were left untransfected, transfected with 2 μg of the HIV-1 Env expression vector, or co-transfected with 2 μg of pCAG-ApoE3-HA vector. Twenty-four hours post-transfection, the cells were untreated (“No inhibitor”) or treated with the lysosomal inhibitors (70 μM leupeptin and 10 μM pepstatin) for an additional 14 h. Then, the cells were lysed and subjected to western blot to analyze the expression of Env. **(C)** MDMs were infected with JR-FL (100 ng/mL) and cultured for 2 days. Then, the cells were untreated or treated with the lysosomal inhibitors (70 μM leupeptin and 10 μM pepstatin) for an additional 8 h, lysed and subjected to western blot to analyze the expression of Env. Since the treatment of MDMs with these inhibitors for 14 h caused a visible toxicity, we employed a shorter (8 h) treatment. In the right panel, the Env levels (quantified by the densitometric analysis) in the treated MDMs are represented as percentages relative to those in the untreated MDMs, and results for MDMs obtained from 3 donors are summarized.

ApoE has several functional domains including the 18 amino acid N-terminal signaling peptide, the LDL-receptor-binding domain (residues 140–160), and the C-terminal lipid-binding domain (residues 202–299) (see Fig 7A). To determine which domain is required for the inhibitory effect of ApoE on HIV-1 Env, we constructed the following ApoE3 deletion mutants: an N-terminal half of ApoE3 (E3N: residues 1–162), an N-terminal half of ApoE3 without the receptor-binding domain (E3NΔ: residues 1–142), a C-terminal half of ApoE3 (E3C: residues 125–299), and a C-terminal half of ApoE3 without the receptor-binding domain (E3CΔ: residues 163–299) (Fig 7A). As shown (Fig 7B), when co-expressed with HIV-1 Env in 293T cells, both E3C and E3CΔ reduced the intracellular Env expression, the degree of which was comparable to that of the full-length ApoE3 whereas both E3N and E3NΔ almost completely lost the inhibitory activity. The LDL-receptor-binding domain was unrelated to the inhibitory activity because both the defective E3N and functional E3C retained the domain. Thus, the C-terminal region including the lipid-binding domain was required for the inhibitory activity of ApoE to HIV-1 Env. Interestingly, all the mutants induced the cytoplasmic localization of Env (S11 Fig), and even the defective ApoE3 mutants (E3N and E3NΔ) co-localized with Env (Fig 7C), suggesting that the inhibitory activity of ApoE to Env was not only due to an interference of the intracellular transport of Env caused by the association between ApoE and Env, but also through an additional mechanism such as a degradation of Env. Indeed, both ApoE and Env were detectable in Rab5-positive early endosome, Rab7-positive late endosome, and also LAMP1-positive lysosome in both the transfected 293T cells (Fig 8A, upper panels). In the quantitative analysis, the co-localization of ApoE/Env with LAMP1 was most obvious (S12 Fig). The localization of Env in LAMP1-positive lysosome was further confirmed in MDMs (Fig 8A, lower panels). Consistent with this, we found that the ApoE-induced suppression of Env expression in the co-expressing 293T cells was almost completely rescued by the treatment with lysosome inhibitors [49] such as leupeptin and pepstatin (Fig 8B). Moreover, these inhibitors tended to enhance Env expression in ApoE siRNA-untransfected MDMs (Fig 8C), suggesting the presence of Env in lysosomes. Finally, when the lysates of 293T cells co-expressing ApoE and HIV-1 Env in the presence of the lysosome inhibitors were incubated with anti-HA antibody to immunoprecipitate the ApoE complex, gp160 was detected in the ApoE immunoprecipitates (S13 Fig), as we observed under the different immunoprecipitation conditions (see Fig 6C). Thus, it was likely that ApoE inhibited HIV-1 production by associating with gp160 and facilitating their degradation in the lysosomes. In summary, our study revealed that ApoE is the HIV-1-inducible inhibitor of viral production and infectivity in macrophages, and suggested that the anti-HIV-1 activity of ApoE is due to the degradation of gp160 Env in the lysosomes and the C-terminal region of ApoE is necessary for the Env degradation.

## Discussion

In this study, we demonstrated that ApoE acts as the HIV-1-inducible inhibitor in primary human macrophages: HIV-1 up-regulates ApoE, which, in turn, inhibits viral production and infectivity by targeting HIV-1 Env in the lysosomes. Of note, HIV-1 selectively up-regulates ApoE among apolipoproteins. ApoE transcription in macrophages is regulated by two multi-enhancers termed ME.1 and ME.2 [78], and has been shown to involve multiple factors including STAT1 [79], glucocorticoid receptor [80], NF-κB [81], AP-1 [81, 82], TGF-β [82], liver X receptors (LXRs) [83], and peroxisome proliferator-activated receptor γ [84]. In addition, the ATP binding cassette transporter ABCA1 modulates ApoE secretion from macrophages [85]. ABCA1, which is defective in Tangier disease, is a key regulator of cholesterol efflux. In cholesterol-loaded macrophages, the activation of LXRs leads to an increased expression of ABCA1, ATP binding cassette transporter G1 (ABCG1) and ApoE to promote cholesterol efflux [83]. However, it remains to be elucidated how HIV-1 selectively induces ApoE, by using these factors. At least, ApoE induction was independent of the IFN system since ApoE is not the ISG (Fig 1E), and also independent of HIV-1 accessary proteins including Nef, Vpr, Vpu and Vif (S2 Fig).

HIV-1 has been associated with ApoA-I [86–88], ApoB [88], and ApoC-III [89]. For instance, ApoA-I, a major protein component of HDL, was reported to inhibit HIV-1 infectivity and HIV-1-induced syncytium formation [86]. ApoA-I was also shown to interact with gp41, a transmembrane subunit of HIV-1 Env, and prevent the insertion of the fusogenic domains into the cellular membrane, resulting in the inhibition of fusion and the entry of HIV-1 into the host cells [87]. Because our results suggested that ApoE, another component of HDL, preferentially binds to the precursor gp160 Env rather than gp120 Env (Fig 6C and S13 Fig), ApoE may interact with gp41 as ApoA-I does. Of interest, although we confirmed the ApoA-I-Env co-localization in 293T cells (S14A Fig), we failed to detect a clear reduction in Env expression by the ApoA-I co-expression (S14B Fig). This might be due to the difference in their inhibitory mechanisms, i.e., the inhibition of HIV-1 entry by Apo-AI [87] and the degradation of Env by ApoE.

Dobson *et al*. [64] and Kelly *et al*. [65] reported the anti-HIV-1 activity of ApoE-derived peptide analogues: they showed that the ApoE-derived peptides acted on target cells whereas our results suggested that ApoE acted on virus-producing cells. On the other hand, Burt et al. reported that purified recombinant ApoE4 proteins enhanced the *in vitro* HIV-1 entry in SupT1-CCR5 cells [55]. Because the frequency of ApoE4 (13–20%) was lower than that of ApoE3 (65–80%), it was possible that we mainly detected the anti-HIV-1 activity of ApoE3 in the knockdown experiments using MDMs, in which all the isoforms were targeted (Figs 2–4). However, the over-expression experiments using 293T cells clearly demonstrated that all the isoforms including ApoE4 had the anti-HIV-1 activity (Fig 5). The lipid-binding domain is critical for its binding to triglyceride-rich lipoproteins [90] and the substitution at glutamic acid 255 in the domain can alter the preference of ApoE4 from VLDL to HDL [90]. In this study, we demonstrated that the C-terminal region including the lipid-binding domain was required for the anti-HIV-1 activity of ApoE3 (Fig 7B). The amino acid sequence of the C-terminal region (residues 125–299) of ApoE3 is identical to that of ApoE4 and the ApoE mutants that had only this region (E3C and E3CΔ) still retained the anti-HIV-1 activity (Fig 7B). Thus, these results may suggest that ApoE plays two different roles in HIV-1 infection: HIV-1 induces ApoE, which inhibits viral production in virus-producing cells but the induced/secreted ApoE enhances viral entry in target cells.

As mentioned above, it was reported that ApoE-derived peptide inhibited HIV-1 entry [64, 65]. The peptide is derived from the LDL-receptor binding domain (LRKLRKRLL; residues 141–149), which apparently contradicts our finding that the LDL-receptor binding domain was dispensable for the anti-HIV-1 activity of ApoE (Fig 7B). However, it should mention that only the tandem repeat peptide (LRKLRKRLLLRKLRKRLL) had a detectable inhibitory activity against HIV-1, which was potentiated by the substitution of its four leucine residues with tryptophan residues [64, 65]. ApoE3 and ApoE4 bind to the LDL receptor with similar affinity as a ligand whereas ApoE2 does not bind to the LDL receptor [17]. Nevertheless, all the isoforms including ApoE2 similarly suppressed HIV-1 Env expression (Fig 5). LDL receptor also serves as the cellular receptor for VSV [91]. However, all the ApoE isoforms failed to suppress VSV-G-pseudotyped viruses (Fig 5H and 5I). These results suggest that the LDL receptor is not involved in the inhibitory effect of ApoE on HIV-1, which is consistent with the finding that the ApoE3 mutant that lacked the LDL-receptor binding domain still suppressed HIV-1 Env expression (Fig 7B).

ApoE as well as several viruses (HIV-1, HSV, Dengue virus, and HCV) interact with heparin sulfate proteoglycans (HSPG) on the cell surface [92]. HSPG participates in HIV-1-cell attachment and virus entry in T cells [93] and macrophages [94]. Similarly, ApoE mediates HCV attachment through an interaction with HSPG [95]. A proteomic and biochemical analysis identified ApoE in purified HIV-1 derived from HIV-1-infected MDMs [66]. Thus, the ApoE-HSPG interaction or ApoE on the HIV-1 virions may regulate the HIV-1 susceptibility to target cells [55]. However, these features of ApoE may not account for its inhibitory effect on HIV-1 observed in this study. HIV-1 infection alters lipid cholesterol status in host cells to stimulate excessive cholesterol accumulation inside cells [95]. This can stimulate the formation of chylomicron, chylomicron remnants, HDL and LDL, and these lipids enhance the secretion of ApoE as it is a vital component of cholesterol efflux in macrophages [16]. Our results suggest that HIV-1 has an additional function to enhance the secretion of ApoE, i.e., the direct up-regulation of transcription of ApoE in macrophages.

Humans have evolved host defense mechanisms with APOBEC3 family including APOBEC3A (A3A), A3B, A3C, A3DE, A3F, A3G, and A3H, while mouse has only APOBEC3. Similarly, only human evolved three ApoE isoforms. All other animals, including the great apes, have a single ApoE isoform that has arginine at the residues equivalent to 112 and 158 [18]. HIV-1 has a counteraction system that uses accessory proteins, such as Vif, Vpu, Vpx and Nef to antagonize host HIV-1 restriction factors, such as APOBEC3G [24–26], Tetherin/BST2 [28, 29], SAMHD1 [30, 31], and SERINC5 [96, 97], respectively. However, the counteractive HIV-1 protein partner for several HIV-1 restriction factors such as TRIM5α, MX-2, and MARCH8 remains unclear. In this study, we demonstrated that all the isoforms of ApoE strongly suppressed the expression of Env regardless of the presence of other HIV-1 proteins including the accessory proteins. Thus, an HIV-1 partner protein, if any, may not be potent in its antagonistic effect on the anti-HIV-1 activity of ApoE3. Also, ApoE is not the ISG, unlike most HIV-1 restriction factors.

A noteworthy finding of our study is that ApoE appears to target HIV-1 Env for the lysosomal degradation (Fig 8), which is consistent with the finding that ApoE is involved in cholesterol homeostasis, lipid antigen presentation and amyloid formation in the endosomal and/or lysosomal pathways [98–100]. Interestingly, recently-identified macrophage-specific HIV-1 restriction factors, such as MARCH8 and DCAF1, were also reported to target HIV-1 Env for the lysosomal degradation [41, 49], although the detailed molecular mechanism is not fully understood. More interestingly, the anti-HIV-1 Env activity of DCAF1 is overcome by HIV-1 Vpr protein [41], and MARCH8 inhibits not only HIV-1 Env but also VSV-G Env more remarkably [49], both of which are not seen with ApoE. Therefore, it will be intriguing to compare how these factors affect the expression level of HIV-1 Env including the responsible molecular pathways, under the same experimental settings. Since the ApoE3 mutants (E3N and E3NΔ) that failed to suppress Env expression still co-localized and presumably associated with Env (Fig 7C), these mutants will be helpful to elucidate how ApoE induces Env degradation. It will be also necessary to clarify to what extent ApoE plays its inhibitory role in HIV-1 infection among donors because the basal expression level of ApoE in macrophages varied among donors (Fig 1B).

In this study, we found that ApoE, when expressed in 293T cells, reduced the level of p24 Gag in the supernatants (Fig 5E, 5F, 5G and 5H). Interestingly, Gag and Env of HIV-1 have been proposed to interact and co-traffic in cells [101]. Thus, it is possible that the reduced p24 in the supernatants was at least in part due to the Gag-Env interaction/co-trafficking. It has been also proposed that the Gag-Env interaction is mediated by the cytoplasmic tail of HIV-1 Env [101], the domain of which is not found in VSV-G Env. Consistent with this, the ApoE expression did not affect the p24 level of viruses pseudotyped with VSV-G Env (Fig 5H).

The current study revealed that ApoE is the HIV-1-inducible inhibitor of viral production and infectivity in macrophages. Mechanistically, ApoE appears to associate with Env and target it for lysosomal degradation. Unlike the lytic infection in T cells, HIV-1-infected macrophages show little viral-induced cytopathic effect and persist in tissue for extended periods with large numbers of infectious particles contained within cytoplasmic vacuoles for unknown mechanisms. Thus, our results will help to understand the molecular basis by which macrophages can maintain the long-term persistent infection of HIV-1.

## Materials and methods

### Ethics statement

Approval for this study was obtained from the Kumamoto University medical ethics committee. Heparinized venous blood was collected from healthy donors after informed consent had been obtained in accordance with the Declaration of Helsinki. All human subjects were adult. The informed consent given was written.

### Preparation of MDMs and HIV-1 infection

Human MDMs were prepared as described previously [102]. Briefly, peripheral blood mononuclear cells were suspended in RPMI 1640 medium containing a low concentration of FBS (1%) to facilitate the adherence of monocytes and seeded into multi-well plates. Monocytes were enriched, by allowing them to adhere to multi-well plates or chamber slides for 1 h, and non-adherent cells were removed by washing with PBS. The adherent monocytes were differentiated into MDMs by culturing with RPMI 1640 supplemented with 10% FBS containing 100 ng/mL rhM-CSF (a gift from Morinaga Milk Industry, Japan). After 3 days, the media were replaced with fresh complete media after through wash with PBS to further remove non-adherent cells, and incubated for another 2 days. The day-5 MDMs were used in the experiments described later.

### HIV-1 infection to MDMs and HIV-1 p24 ELISA

Recombinant HIV-1 was prepared as described previously [102]. Briefly, HEK293A cells (Invitrogen, Carlsbad, CA, USA) cultured in DMEM supplemented with 10% FBS were used as viral producer cells. The cells were seeded into 12-well tissue culture plates and transfected with the HIV-1 molecular clone (pJR-FL, provided by Dr. Y. Koyanagi, Kyoto University, Kyoto, Japan) using Lipofectamine 2000 reagent (Invitrogen). After 6 h of transfection, culture media were replaced with fresh media, and the cells were cultured for an additional 48 h. Then, the supernatants containing recombinant viruses were clarified by centrifugation, analyzed for their HIV-1 p24 concentrations by ELISA (MBL, Nagoya, Japan), and stored at -70°C before use. HIV-1 infection was performed as described previously [102]. MDMs were incubated with 200 μL (for 24-well plate) or 400 μL (for 2-well chamber slide) of the supernatants of HEK293A cells containing HIV-1 (100 ng/mL of p24 unless otherwise stated) for 2 h at 37°C. Then, the cells were washed twice with PBS to remove any unbound viruses, cultured with media containing rhM-CSF and subjected to the experiments including microarray, western blot and real-time RT-PCR.

### Microarray

Microarray analysis was performed as described previously [103]. Total RNA prepared from MDMs was biotin-labeled using a GeneChip 3’IVT express kit (Affymetrix), and microarray analysis was performed at TaKaRa-Bio using high-density Affymetrix GeneChip oligonucleotide arrays (Human Genome U133 Plus 2.0). Data was analyzed using GeneSpring 14.5 software (Agilent Technologies). Microarray data have been deposited in the National Center for Biotechnology Information Gene Expression Omnibus (GSE71290; http://www.ncbi.nlm.nih.gov/geo).

### Western blot

Western blot was performed as described previously [104–106]. MDMs or transfected 293T cells were lysed in buffer containing 50 mM Tris-HCl (pH 8.0), 150 mM NaCl, 4 mM EDTA, 1% Nonidet (N) P-40, 0.1% sodium dodecyl sulfate (SDS), 1 mM dithiothreitol (DTT) and 1 mM phenyl-methylsulfonyl fluoride (PMSF). Supernatants from these lysates were subjected to SDS-polyacrylamide gel electrophoresis and transferred to Immobilon-P PVDF transfer membranes. The membranes were then incubated with the following primary antibodies: anti-ApoE (EP1347Y; Abcam), anti-apolipoprotein B (AB742; EMD Millipore), anti-HIV-1 p24 rabbit polyclonal antibody (65–004; Bioacademia, Japan), anti-HIV-1 p24 mouse monoclonal antibody (A2-851-100; Icosagen, Estonia), anti-HIV-1 Env (KD-247) [76], anti-VSV Glycoprotein (P5D4; Sigma), anti-HA (HA-7; Sigma), and anti-*β*-actin (AC-15; Sigma). After the incubation with HRP-labeled secondary antibodies, proteins were visualized using Western Lightning Plus-ECL (PerkinElmer) and an Amersham Imager 600 imaging system (GE Healthcare). In a selected experiment, the intensity of bands was quantified using the ImageJ v.1.50i software.

### Real-time RT-PCR

Real-time RT-PCR was performed as described previously [106]. MDMs were treated with 1,000 U/mL of IFN-α (Sigma) for 24 h of IFN-α in selected experiments. Alternatively, MDMs were transfected with siRNAs (see below). Total RNA was isolated using an RNeasy mini kit (QIAGEN), and cDNAs were synthesized using an oligo(dT)_12-18_ primer and M-MLV reverse transcriptase (Invitrogen). Then, real-time RT-PCR was performed with *ApoE*, *MX2* or *β-actin* primer sets and SYBR Premix Ex Taq II (TaKaRa-Bio) using a LightCycler Nano (Roche) with 35 cycles. Primer sequences are as follows: 5’-AGCTGGTTCGAGCCCCTGGTG-3’ (forward) and 5’-TCAGTGATTGTCGCTGGGCAC-3’ (reverse) for *ApoE*, 5’-ATGTCTAAGGCCCACAAGCCT-3’ (forward) and 5’-TGGCACTGTGCCGAATGGCGG-3’ (reverse) for *MX2*, 5’-TGACGGGGTCACCCACACTG-3’ (forward) and 5’-AAGCTGTAGCCGCGCTCGGT-3’(reverse) for *β-actin*.

### RNA interference

Knockdown of ApoE in MDMs was performed using Lipofectamine RNAiMAX reagent (Invitrogen) according to the manufacturer’s instructions. MDMs cultured on 24-well plates (approximately 1x10^5^ cells/well) were cultured with antibiotic-free media for 1 day, and then transfected with 10 pmol/well of siRNA using 1.5 μL/well of Lipofectamine RNAiMAX. Both the control siRNAs (non-targeting siRNA pool; D-001206-13, non-targeting siRNA #1; D-001210-01) and the ApoE-specific siRNAs (ApoE siRNA pool; M-006470-00, ApoE siRNA #1; D-006470-01, ApoE siRNA #2; D-006470-02) were purchased from Dharmacon. After 6 h of transfection, the culture media were replaced with fresh media, and cultured for another 2 days. The knockdown efficiency was assessed by western blot. MDMs transfected were also subjected to the experiments including flow cytometry, MTT assay and HIV-1 infection.

### Flow cytometry and cell viability

MDMs were transfected with the control or ApoE siRNA and cultured for 2 days. Then, MDMs were detached from plates using enzyme-free cell dissociation buffer (Life Technologies) and analyzed for the cell surface expression of CD4 or CCR5 by flow cytometry on FACSVerse (BD Biosciences) using FlowJo software (Tree Star), as described previously [102]. The following antibodies were used: APC-labeled anti-CD4 (RPA-T4; BioLegend) and FITC-labeled anti-CCR5 (HEK/1/85a; BioLegend). Also, MDMs infected with HIV-1 were fixed in 1% paraformaldehyde (Sigma), permeabilized with 0.1% saponin (Sigma), stained with FITC-labeled anti-HIV-1 p24 (KC57; Coulter), and analyzed by flow cytometry [103]. Cell viability was assessed using MTT reagent as described previously [103]. The absorbance of the wells was measured at 595 nm.

### Viral infectivity assay

The viral infectivity was assessed using TZM-bl cells (NIH AIDS Research & Reference Program), as described previously [102]. The cells (1x10^5^ cells/well) were seeded onto 24-well plates and infected with serially diluted viruses normalized for the concentration of p24 proteins. At 24 h post-infection, firefly luciferase activity was measured with a LB9507 luminometer (Berthold, Germany).

### Immunofluorescence

Immunofluorescence was performed as described previously [107]. 293T cells (3x10^4^ cells/well) were cultured in 2-well chamber slide and co-transfected with 100 ng pJR-FL and 100 ng pCAG-ApoE3-HA. Cells were fixed in 3.6% formaldehyde in PBS and permeabilized in 0.1% NP-40 in PBS at room temperature. The cells were incubated with anti-HIV-1 p24 (65–005; Bioacademia, Japan), anti-HA (HA-7), anti-HIV-1 Env (KD-247), anti-Rab5 (C8B1; Cell Signaling Technology, Danvers, MA), anti-Rab7 (D95F2; Cell Signaling Technology), anti-LAMP1 (ab24170, Abcam, Cambridge, UK), anti-CD14 (M-305; Santa Cruz Biotechnology), and/or anti-ApoE (EP1347Y; Abcam) antibody at a 1:300 dilution in PBS containing 3% BSA. Cells were then stained with Alexa Fluor 488 goat anti-Human IgG (A11013), Alexa Fluor 594 donkey anti-mouse IgG (A21203) and/or Alexa Fluor 647 donkey anti-rabbit IgG antibody (A315719) (all from Molecular probe) at a 1:300 dilution in PBS containing 3% BSA. Nuclei were stained with DAPI. Following extensive washing in PBS, the cells were mounted on slides using a mounting media of SlowFade Gold anti-fade reagent (Invitrogen). Samples were viewed under a confocal laser-scanning microscope (FV1200; Olympus, Tokyo, Japan). Image processing was performed using the FV Viewer ver. 4.1 software (Olympus). The cellular localization of target proteins including Env was quantified using ImageJ software. The co-localization of different target proteins was also quantified using the built in coloc2 plugin. In brief, the Manders overlap coefficient (MOC), as a measure of the colocalization between the different fluorescent signals in each channel, was calculated.

### Transfection into 293T cells

Transient transfection was performed using 293T cells (Invitrogen) and TransIT-LT1 (Mirus) according to the manufacturer’s protocol, as described previously [107]. The cells were cultured with DMEM supplemented with 10% FBS.

### ApoE and its mutant plasmids

The ApoE expression vectors (pCAG-ApoE2-HA, pCAG-ApoE3-HA, and pCAG-ApoE4-HA) [58] were used. To construct pcDNA3-ApoE3-HA, pcDNA3-ApoE3N-HA, pcDNA3-ApoE3NΔ-HA, and pcDNA3-ApoE3C-HA, each ApoE3 DNA fragment was amplified from pCAG-ApoE3 [58] by PCR using Tks Gflex DNA polymerase (TaKaRa-Bio) and the following pairs of primers: 5’-CCGGGATCCAAGATGAAGGTTCTGTGGGCT-3’ (Forward) and 5’-CGCTCGAGTCAGGCATAGTCAGGCACGTCATAAGGATAGTGATTGTCGCTGGGCACA-3’ (Reverse) for ApoE3-HA (E3), 5’-CCGGGATCCAAGATGAAGGTTCTGTGGGCT-3’ (Forward) and 5’-CGCTCGAGTCAGGCATAGTCAGGCACGTCATAAGGATAGTACACTGCCAGGCGCTTC-3’ (Reverse) for ApoE3N (E3N), 5’-CCGGGATCCAAGATGAAGGTTCTGTGGGCT-3’ (Forward) and 5’-CGCTCGAGTCAGGCATAGTCAGGCACGTCATAAGGATAGTGGGAGGCGAGGCGACAC-3’ (Reverse) for ApoE3NΔ (E3NΔ), 5’-CCGGGATCCAAGATGCTCGGCCAGAGCACC-3’ (Forward) and 5’-CGCTCGAGTCAGGCATAGTCAGGCACGTCATAAGGATAGTGATTGTCGCTGGGCACA-3’ (Reverse) for ApoE3C (E3C), and 5’-CCGGGATCCAAGATGCAGGCCGGGGCCCGCGAG-3’ (Forward) and 5’-CGCTCGAGTCAGGCATAGTCAGGCACGTCATAAGGATAGTGATTGTCGCTGGGCACA-3’ (Reverse) for ApoE3CΔ (E3CΔ). The amplified PCR products were subcloned into a *Bam*H1-*Xho*I site of pcDNA3.

### HIV-1 molecular clones and Env plasmids

HIV-1 molecular clones used for transfection were as follows: pJR-FL [67], pAD8 [70], pNL4-3 [71], and pR9 [72, 73]. In a selected experiment, the pR9 mutant lacking Env expression (pR9ΔEnv) was used [72, 73]. Also, in selected experiments, the HIV-1 JR-FL Env expression plasmid pJR-FL Env [108] or VSV-G Env expression plasmid pMD.2G [109, 110] were used.

### Collection and analysis of released virions

Released HIV-1 virions were collected by centrifugation of the culture supernatants from transfected 293T cells at 20,000 x g for 2 h at 4 °C. The pellets containing virions were dissolved in the lysis buffer and then subjected to western blot.

### Immunoprecipitation

Immunoprecipitation was performed as described previously [111]. Transfected 293T cells were lysed in the buffer containing 10 mM Tris-HCl (pH 8.0), 150 mM NaCl, 4 mM EDTA, 0.1% NP-40, 10 mM NaF, 1 mM DTT, and 1 mM PMSF. The lysates were pre-cleared with Protein-G-Sepharose (GE Healthcare), and incubated with anti-HA antibody (3F10; Roche) at 4 °C for 1 h. After the incubation with Protein-G-Sepharose for 1 h, the resin was washed five times with the lysis buffer. Proteins eluted were then subjected to western blot.

### Lysosomal inhibitors

MDMs or transfected 293 T cells were incubated with the lysosomal inhibitors (purchased from Peptide Institute, Osaka, japan), leupepetin (70 μM) and pepstatin (10 μM). Leupeptin and pepstatin was dissolved in H_2_O and DMSO, respectively, and the same volumes of H_2_O and DMSO were used as vehicle controls.

### Statistical analysis

The statistical significance of the inter-sample differences was determined using the paired Student’s *t* test. *p* values of < 0.05 were considered significant.

## Supporting information

S1 Fig Effect of HIV-1 infection on ApoE expression in MDMs. MDMs (2 donors) were infected with HIV-1 JR-FL (100 ng/mL p24) by using the supernatants of HEK293A cells transfected with the HIV-1 molecular clone as a source of viruses (“HIV-1”). To confirm that the ApoE induction was due to HIV-1 infection, MDMs were also incubated with the same volume of the supernatants of HEK293A cells transfected with the empty vector pUC119 (“Mock”). Those MDMs were then cultured for 1, 2, 3, 5, or 8 days, lysed, and subjected to western blot to analyze the expression of ApoE, or ApoB as a reference, using anti-ApoB antibody (AB742; EMD Millipore). HuH-7, the human hepatoma cell line (a gift from Dr. N. Kato, Okayama University, Okayama, Japan), was used as a positive control fro ApoB expression (right-most lane). MDMs were also analyzed for the expression of HIV-1 p24 to verify the viral replication. Anti-β-actin blot was used as a loading control. The lysates of MDMs were also prepared immediately before HIV-1 infection as a control (“0 dpi”).
(EPS)

S2 Fig Effects of infection of HIV-1 mutant viruses on ApoE expression in MDMs. **(A)** MDMs were infected with the AD8 strain of HIV-1 (100 ng/mL p24) by using the supernatants of 293T cells transfected with the HIV-1 molecular clones as a source of viruses, and cultured for 5 days. In addition to the wild-type (WT), Nef-deficient (ΔNef), Vpr-deficient (ΔVpr), Vpu-deficient (ΔVpu) and Vif-deficient (ΔVif) mutant viruses were used (Schubert U, Clouse KA, Strebel K. Augmentation of virus secretion by the human immunodeficiency virus type 1 Vpu protein is cell type independent and occurs in cultured human primary macrophages and lymphocytes. J. Virol. 1995; 69: 7699–7711). The control uninfected MDMs were prepared by culturing with media for 5 days. Those MDMs were lysed and subjected to western blot to analyze the expression of ApoE as well as HIV-1 Gag (p24 and p55). Anti-β-actin blot was used as a loading control. As shown, the mutant viruses such as AD8ΔVif, the growth of which was weaker than that of the wild-type viruses, still up-regulated ApoE in MDMs. Thus, the lower replication level might be enough for ApoE induction. Alternatively, the attachment of viruses to the cell surface receptors or viral entry might trigger ApoE induction. **(B)** MDMs were infected with the WT or ΔVif viruses as in (A). Three different preparations of viral stock were used. MDMs were then cultured for 5 days, lysed, and subjected to western blot to analyze the expression of ApoE. The blots with shorter (1 min) and longer (3 min) exposures are shown. The difference in ApoE level between WT and ΔVif was detectable in the preparation-3 as in (A), but ΔVif virus of the preparations 1 and 2 did not necessarily up-regulated ApoE more potently than WT virus.
(EPS)

S3 Fig Effect of HIV-1 infection on ApoE expression in CD4^+^ T cells. **(A)** MT-4 cells (5x10^6^ cells) were left uninfected or infected with the NL4-3 strain of HIV-1 (total 200 ng of p24), cultured for 3 days, lysed, and subjected to western blot to analyze the expression of ApoE or p24 (to verify the viral replication). Anti-β-actin blot was used as a loading control. **(B)** Peripheral blood mononuclear cells were seeded onto dishes to allow monocytes to adhere. The non-adherent cells containing CD4^+^ T cells (3 donors) were activated with PHA (50 μg/mL; Sigma) and rhIL-2 (Prospec) for 2 days, and the cultured for 24 h with rhIL-2 alone. Then, they (5x10^6^ cells) were left uninfected or infected with NL4-3 (total 200 ng of p24), cultured for 3 days, and analyzed as in (A).
(EPS)

S4 Fig Effect of ApoE knockdown on the formation of HIV-1-infected multi-nucleated MDMs. MDMs were transfected with either ApoE-targeting siRNA (“si-ApoE”) or non-targeting siRNA as a control (“si-Cr”), which is a mixture of 4 siRNAs (“4-pool”), cultured for 2 days, infected with HIV-1 JR-FL (100 ng/mL p24) for another 2 days, and stained with DAPI to identify multi-nucleated MDMs (indicated by yellow arrowheads). As we recently showed (Hashimoto M, Bhuyan F, Hiyoshi M, Noyori O, Nasser H, Miyazaki M, et al. Potential role of the formation of tunneling nanotubes in HIV-1 spread in macrophages. J. Immunol. 2016; 196:1832–1841), the nuclei were arranged in a circular pattern in HIV-1-infected fused MDMs. The numbers of the multi-nucleated MDMs were also quantified (see Fig 2F). Data shown are representative of experiments obtained from 2 different donors with similar results.
(EPS)

S5 Fig Effect of ApoE knockdown on the localization of Env in MDMs. **(A-C)** MDMs were transfected with either pooled non-targeting siRNAs (upper panels) or ApoE siRNA #1 (lower panels), cultured for 2 days, infected with HIV-1 JR-FL (100 ng/mL p24) and co-stained with anti-Env antibodies (green), anti-CD14 antibodies (red) and DAPI (blue) at 2 dpi. The signal of Env and CD14 within the yellow frames (A) was quantified in (B) and (C).
(EPS)

S6 Fig Expression of ApoE in various cell lines including 293T cells. The expression of ApoE in the indicated cell lines was analyzed by western blot. Anti-β-actin blot was used as a loading control. U937: a human monocytic cell line.
(EPS)

S7 Fig Effect of exogenous expression of ApoE on Env expression in 293T cells, and the levels of ApoE in the transfected 293T cells and HIV-1-infected MDMs. The 293T cells were co-transfected with 2 μg of HIV-1 molecular clone (JR-FL) and the indicated amounts of HA-tagged ApoE3 expression vector, cultured for 2 days, lysed, and subjected to western blot to analyze the expression of Env and p24. ApoE expression was also verified. Anti-β-actin blot was used as a loading control. In order to show the level of the expression of ApoE in 293T cells, uninfected MDMs and MDMs infected with JR-FL for 8 days (see donor 2 in S1 Fig) were added as references.
(EPS)

S8 Fig Effect of exogenous expression of ApoE on viral infectivity of HIV-2. The 293T cells were transfected with 2 μg of HIV-2 molecular clone (ROD10) (Chen CY, Shingai M, Welbourn S, Martin MA, Borrego P, Taveira N, et al. Antagonism of BST-2/Tetherin is a conserved function of the Env glycoprotein of primary HIV-2 isolates. J. Virol. 2016; 90: 11062–11074.), or co-transfected with 2 μg of HA-tagged ApoE3 expression vector. The supernatants of the transfected 293T cells were collected and analyzed for their levels of p24 concentration by ELISA (upper panel). Then, the infectivity of the produced viruses (lower panel) was measured (lower panel). TZM-bl indicator cells were infected with HIV-2 ROD10 (input p24: 0.5 ng/mL), and at 24 h of post-infection, the viral infectivity was measured by luciferase assays. The infectivity of the viruses produced by ApoE3 co-transfected cells are represented as percentages relative to that by empty vector-transfected cells. Error bars indicate standard deviations of triplicate assays.
(EPS)

S9 Fig Effects of exogenous expression of ApoE on the expression of Env and p24 in the absence of other viral proteins in 293T cells. **(A)** The 293T cells were transfected with 2 μg of the HIV-1 Env expression vector (pJR-FL Env), or co-transfected with 2 μg of the indicated HA-tagged ApoE expression vector, cultured for 2 days, lysed, and subjected to western blot to analyze the expression of Env. ApoE expression was verified using anti-HA antibody. Anti-β-actin blot was used as a loading control. **(B)** The 293T cells were transfected with 2 μg of an HIV-1 p24 expression vector, or co-transfected with 2 μg of the indicated HA-tagged ApoE expression vector, cultured for 2 days, lysed, and subjected to western blot to analyze the expression of p24. The p24 expression vector was prepared as follows: the DNA fragment encoding p24 was amplified from pCMVΔR8.74 (Zufferey R, Nagy D, Mandel RJ, Naldini L, Trono D. Multiply attenuated lentiviral vector achieves efficient gene delivery in vivo. Nat Biotechnol. 1997; 15: 871–875) by PCR using KOD-Plus DNA polymerase (TOYOBO, Osaka, Japan) and the following primer pair: 5’-CGGGATCCAAGATGCCTATAGTGCAGAACATCCAG-3’ (forward), 5’-CCGGCGGCCGCTTACAAAACTCTTGCTTTA-3’ (reverse), and then the amplified PCR product was sub-cloned into the *Bam*H1-*Not*I site of pcDNA3 vector. **(C)** The 293T cells were transfected with 2 μg of the AD8 Env expression vector (pNLA1-AD8) or HIV-2 (ROD10) Env expression vector (pCM10-Env-HA) (Schubert U, Clouse KA, Strebel K. Augmentation of virus secretion by the human immunodeficiency virus type 1 Vpu protein is cell type independent and occurs in cultured human primary macrophages and lymphocytes. J. Virol. 1995; 69: 7699–7711), or co-transfected with 2 μg of the ApoE3-HA expression vector, cultured for 2 days, lysed, and subjected to western blot to analyze the expression of Env.
(EPS)

S10 Fig Subcellular localization of HIV-1 Env in the absence or presence of ApoE3 in 293T cells. The 293T cells were transfected with 100 ng of the HIV-1 molecular clone JR-FL or co-transfected with 100 ng of the HA-tagged ApoE3 expression vector in a 2-well chamber slide. Twenty-four hours post-transfection, the cells were stained to detect Env signal (see Fig 6A). The cells were randomly selected (10 cells for each group) and analyzed for their distribution of Env signal. The positions of the plasma membrane are indicated by arrows.
(EPS)

S11 Fig Subcellular localization of HIV-1 Env in the presence of ApoE mutants in 293T cells. The 293T cells were co-transfected with 100 ng of the HIV-1 molecular clone JR-FL and 100 ng of the indicated ApoE3 mutant expression vector. Twenty-four hours post-transfection, the cells were stained to detect Env signal (see Fig 7C). The cells were randomly selected (5 cells for each group) and analyzed for their distribution of Env signal. The positions of the plasma membrane are indicated by arrows.
(EPS)

S12 Fig Quantification of the presence of HIV-1 Env and ApoE in endosomes and lysosomes in 293T cells. **(A-C)** The 293T cells were co-transfected with 100 ng of the HIV-1 molecular clone JR-FL and 100 ng of pCAG-ApoE3-HA in a 2-well chamber slide. Twenty-four hours post-transfection, the cells were co-stained to detect the signal of Env, ApoE, Rab5, Rab7, or LAMP1 (see Fig 8A, upper panels). The subcellular distribution of Env/ApoE and either Rab5 (A), Rab7 (B), or LAMP1 (C) was analyzed by measuring the Mander’s overlap coefficient using the coloc 2 ImageJ software plugin.
(EPS)

S13 Fig The association between ApoE and gp160 Env in 293T cells treated with lsosomal inhibitors. The 293T cells were co-transfected with 4 μg of the HIV-1 molecular clone JR-FL and 4 μg of the indicated HA-tagged ApoE expression vector (either ApoE3 or ApoE4). Twenty-four hours post-transfection, the cells were treated with the lysosomal inhibitors (70 μM leupeptin and 10 μM pepstatin) for an additional 14 h. Then, the cells were lysed and incubated with the anti-HA antibody (to precipitate the ApoE complex), and the ApoE immunoprecipitates (IP) were analyzed for the presence of Env by western blot (upper right). The level of Env in the total cell lysates is also shown (upper left, “input”). The arrowheads indicate gp160 and gp120 of Env. In the bar graph, the gp160 or gp120 levels (quantified by the densitometric analysis) in the ApoE immunoprecipitates are represented as percentages relative to those in total cell lysates (”Recovery in ApoE-IP”), and results obtained from 3 independent experiments are summarized. *p < 0.0001.
(EPS)

S14 Fig Effect of exogenous expression of ApoA-I on the localization and expression of HIV-1 Env in 293T cells. **(A)** The 293T cells were co-transfected with 100 ng of the HIV-1 molecular clone (pJR-FL) and 100 ng of an ApoA-I expression vector (pcDNA3-ApoA-I-HA) in a 2-well chamber slide. Twenty-four hours post-transfection, cells were stained with anti-HIV-1 Env (KD-247) and anti-HA (HA-7) antibodies, and then visualized with AlexaFluor488 goat anti-Human IgG and AlexaFluor594 donkey anti-mouse IgG antibodies. Nuclei were stained with DAPI. Images were visualized using a confocal laser scanning microscopy. **(B)** The 293T cells were transfected with the 2 μg of pJR-FL, or co-transfected with 2 μg of pcDNA3-ApoA-I-HA, cultured for 2 days, lysed, and subjected to western blot to analyze the expression of Env. ApoA-I expression was verified using anti-HA antibody. Anti-β-actin blot was used as a loading control. The ApoA-I expression vector was prepared as follows: total RNA from HuH-7 was reverse-transcribed with oligo(dT)_12-18_ primer (Invitrogen), and the cDNA fragment encoding ApoA-I was amplified by PCR using KOD-Plus DNA polymerase and the following primer pair: 5’-CCGGGATCCAAGATGAAAGCTGCGGTGCTG-3’ (forward) and 5’-CCGGCGGCCGCTCAGGCATAGTCAGGCACGTCATAAGGATACTGGGTGTTGAGCTTCTT -3’ (reverse). The amplified DNA fragment was sub-cloned into the *Bam*H1-*Not*I site of pcDNA3 vector.
(EPS)

## References

[1] Mahley RW. Apolipoprotein E: cholesterol transport protein with expanding role in cellbiology.Science. 1988; 240: 622–630. 3283935 10.1126/science.3283935

[2] Mahley RW, Rall SC Jr. Apolipoprotein E: far more than a lipid transport protein. Annu Rev Genomics Hum Genet. 2000; 1: 507–537. 10.1146/annurev.genom.1.1.507 11701639 10.1146/annurev.genom.1.1.507

[3] Mahley RW, Weisgraber KH, Huang Y. Apolipoprotein E: structure determines function, from atherosclerosis to Alzheimer’s disease to AIDS. J Lipid Res. 2009; 50 Suppl: S183–188. 19106071 10.1194/jlr.R800069-JLR200 PMC2674716

[4] Mahley RW. Apolipoprotein E: from cardiovascular disease to neurodegenerative disorders. J Mol Med. 2016; 94: 739–746. 10.1007/s00109-016-1427-y 27277824 10.1007/s00109-016-1427-y PMC4921111

[5] Mahley RW, Weisgraber KH, Huang Y. Apolipoprotein E4: a causative factor and therapeutic target in neuropathology, including Alzheimer’s disease. Proc Natl Acad Sci USA. 2006; 103: 5644–5651. 10.1073/pnas.0600549103 16567625 10.1073/pnas.0600549103 PMC1414631

[6] Mahley RW, Innerarity TL, Rall SC Jr, Weisgraber KH. Plasma lipoproteins: apolipoprotein structure and function. J Lipid Res. 1984; 25: 1277–1294. 6099394

[7] Zannis VI, Breslow JL, Utermann G, Mahley RW, Weisgraber KH, Havel RJ, et al Proposed nomenclature of apoE isoproteins, apoE genotypes, and phenotypes. J Lipid Res. 1982; 23: 911–914. 7130859

[8] Rall SC Jr, Weisgraber KH, Mahley RW. Human apolipoprotein E. The complete amino acid sequence. J Biol Chem. 1982; 257: 4171–4178. 7068630

[9] McLean JW, Elshourbagy NA, Chang DJ, Mahley RW, Taylor JM. Human apolipoprotein mRNA. cDNA cloning and nucleotide sequencing of a new variant. J Biol Chem. 1984; 259: 6498–6504. 6327682

[10] Smit M, van der Kooil-Meijs E, Frants RR, Havekes L, Klasen EC. Apolopoprotein gene cluster on chromosome 19. Definite localization of the APOC2 gene and the polymorphic Hpa I site associated with type III hyperlipoproteinemia. Hum Genet. 1988; 78: 90–93. 2892779 10.1007/BF00291243

[11] Myklebost O, Rogne S. A physical map of the apolipoprotein gene cluster on human chromosome 19. Hum Genet. 1988; 78: 244–247. 2894348 10.1007/BF00291670

[12] Elshourbagy NA, Liao WS, Mahley RW, Taylor JM. Apolipoprotein E mRNA is abundant in the brain and adrenals, as well as in the liver, and is present in other peripheral tissues of rats and marmosets. Proc Natl Acad Sci USA. 1985; 82: 203–207. 3918303 10.1073/pnas.82.1.203 PMC397000

[13] Blue ML, Williams DL, Zucker S, Khan SA, Blum CB. Apolipoprotein E synthesis in human kidney, adrenal gland, and liver. Proc Natl Acad Sci U S A. 1983; 80: 283–287. 6572003 10.1073/pnas.80.1.283 PMC393357

[14] Basu SK, Brown MS, Ho YK, Havel RJ, Goldstein JL. Mouse macrophages synthesize and secrete a protein resembling apolipoprotein E. Proc Natl Acad Sci USA. 1981; 78: 7545–7549. 6950395 10.1073/pnas.78.12.7545 PMC349305

[15] Basu SK, Ho YK, Brown MS, Bilheimer DW, Anderson RG, Goldstein JL. Biochemical and genetic studies of the apoprotein E secreted by mouse macrophages and human monocytes. J Biol Chem. 1982; 257: 9788–9795. 6286633

[16] Kockx M, Jessup W, Kritharides L. Regulation of endogenous apolipoprotein E secretion by macrophages. Arterioscler Thromb Vasc Biol. 2008; 28:1060–1067. 10.1161/ATVBAHA.108.164350 18388328 10.1161/ATVBAHA.108.164350

[17] Weisgraber KH, Innerarity TL, Mahley RW. Abnormal lipoprotein receptor-binding activity of the human E apoprotein due to cycteine-arginine intercharge at a single site. J Biol Chem. 1982; 257: 2518–2521. 6277903

[18] Mclntosh AM, Bennett C, Dickson D, Anestis SF, Watts DP, Webster TH, et al The apolipoprotein E (APOE) gene appears functionally monomorphic in chimpanzees (Pan troglodytes). PLoS One. 2012; 7: e47760 10.1371/journal.pone.0047760 23112842 10.1371/journal.pone.0047760 PMC3480407

[19] Meltzer MS, Gendelman HE. Mononuclear phagocytes as targets, tissue reservoirs, and immunoregulatory cells in human immunodeficiency virus disease. Curr Top Microbiol Immunol. 1992; 181: 239–263. 1424782 10.1007/978-3-642-77377-8_9

[20] Kedzierska K, Crowe SM. The role of monocytes and macrophages in the pathogenesis of HIV-1 infection. Curr Med Chem. 2002; 9: 1893–1903. 12369874 10.2174/0929867023368935

[21] Bergamaschi A, Pancino G. Host hindrance to HIV-1 replication in monocytes and macrophages. Retrovirology. 2010; 7: 31 10.1186/1742-4690-7-31 20374633 10.1186/1742-4690-7-31 PMC2868797

[22] Doyle T, Goujon C, Malim MH. HIV-1 and interferons: who’s interfering with whom? Nat Rev Microbiol. 2015; 13: 403–413. 10.1038/nrmicro3449 25915633 10.1038/nrmicro3449 PMC7768976

[23] Goujon C, Malim MH. Characterization of the alfa interferon-induced postently block to HIV-1 infection in primary human macrophages and T cells. J Virol. 2010; 84: 9254–9266. 10.1128/JVI.00854-10 20610724 10.1128/JVI.00854-10 PMC2937661

[24] Sheehy AM, Gaddis NC, Choi JD, Malim MH. Isolation of a human gene that inhibits HIV-1 infection and is suppressed by the viral Vif protein. Nature. 2002; 418: 646–650. 10.1038/nature00939 12167863 10.1038/nature00939

[25] Mangeat B, Turelli P, Caron G, Friedli M, Perrin L, Trono D. Broad antiretroviral defence by human APOBEC3G through lethal editing of nascent reverse transcripts. Nature. 2003; 424: 99–103. 10.1038/nature01709 12808466 10.1038/nature01709

[26] Harris RS, Bishop KN, Sheehy AM, Craig HM, Petersen-Mahrt SK, Watt IN, et al DNA deamination mediates innate immunity to retroviral infection. Cell. 2003; 113: 803–809. 12809610 10.1016/s0092-8674(03)00423-9

[27] Stremlau M, Owens CM, Perron MJ, Kiessling M, Autissier P, Sodroski J. The cytoplasmic body component TRIM5alpha restricts HIV-1 infection in Old World monkeys. Nature. 2004; 427: 848–853. 10.1038/nature02343 14985764 10.1038/nature02343

[28] Neil SJ, Zang T, Bieniasz PD. Tetherin inhibits retrovirus release and is antagonized by HIV-1 Vpu. Nature. 2008; 451: 425–430. 10.1038/nature06553 18200009 10.1038/nature06553

[29] van Damme N, Goff D, Katsura C, Jorgenson RL, Mitchell R, Johnson MC, et al. The interferon-induced protein BST-2 restricts HIV-1 release and is downregulated from the cell surface by the viral Vpu protein. Cell Host Microbe. 2008; 3: 245–252. 10.1016/j.chom.2008.03.001 18342597 10.1016/j.chom.2008.03.001 PMC2474773

[30] Laguette N, Sobhian B, Casartelli N, Ringeard M, Chable-Bessia C, Ségéral E, et al SAMHD1 is the dendritic- and myeloid-cell-specific HIV-1 restriction factor counteracted by Vpx. Nature. 2011; 474: 654–657. 10.1038/nature10117 21613998 10.1038/nature10117 PMC3595993

[31] Hrecka K, Hao C, Gierszewska M, Swanson SK, Kesik-Brodacka M, Srivastava S, et al Vpx relieves inhibition of HIV-1 infection of macrophages mediated by the SAMHD1 protein. Nature. 2011; 474: 658–661. 10.1038/nature10195 21720370 10.1038/nature10195 PMC3179858

[32] Goujon C, Moncorgé O, Bauby H, Doyle T, Ward CC, Schaller T, et al Human MX2 is an interferon-induced post-entry inhibitor of HIV-1 infection. Nature. 2013; 502: 559–562. 10.1038/nature12542 24048477 10.1038/nature12542 PMC3808269

[33] Liu Z, Pan Q, Ding S, Qian J, Xu F, Zhou J, et al The interferon-inducible MxB protein inhibits HIV-1 infection. Cell Host Microbe. 2013; 14: 398–410. 10.1016/j.chom.2013.08.015 24055605 10.1016/j.chom.2013.08.015

[34] Kane M, Yadav SS, Bitzegeio J, Kutluay SB, Zang T, Wilson SJ, et al MX2 is an interferon-induced inhibitor of HIV-1 infection. Nature. 2013; 502: 563–566. 10.1038/nature12653 24121441 10.1038/nature12653 PMC3912734

[35] Simon V, Bloch N, Landau NR. Intrinsic host restrictions to HIV-1 and mechanisms of viral escape. Nat Immunol. 2015; 16: 546–553. 10.1038/ni.3156 25988886 10.1038/ni.3156 PMC6908429

[36] Peng G, Lei KJ, Jin W, Greenwell-Wild T, Wahl SM. Induction of APOBEC3 family proteins, a defensive maneuver underlying interferon-induced anti-HIV-1 activity. J Exp Med. 2006; 203: 41–46. 10.1084/jem.20051512 16418394 10.1084/jem.20051512 PMC2118075

[37] Peng G., Greenwell-Wild T., Nares S., Jin W., Lei K.J., Rangel Z.G. et al Myeloid differentiation and susceptibility to HIV-1 are linked to APOBEC3 expression. Blood. 2007; 110: 393–400. 10.1182/blood-2006-10-051763 17371941 10.1182/blood-2006-10-051763 PMC1896122

[38] Anderson J, Akkina R. TRIM5α_rh_ expression restricts HIV-1 infection in lentiviral vector-transduced CD34^+^-cell-derived macrophages. Mol Ther. 2005; 12: 687–696. 10.1016/j.ymthe.2005.07.291 16081321 10.1016/j.ymthe.2005.07.291

[39] Bergamaschi A, David A, Le Rouzic E, Nisole S, Barré-Sinoussi F, Pancino G. The CDK inhibitor p21Cip1/WAF1 is induced by FcgammaR activation and restricts the replication of human immunodeficiency virus type 1 and related primate lentiviruses in human macrophages. J Virol. 2009; 83:12253–12265. 10.1128/JVI.01395-09 19759136 10.1128/JVI.01395-09 PMC2786717

[40] Allouch A, David A, Amie SM, Lahouassa H, Chartier L, Margottin-Goguet F, et al p21- mediated RNR2 repression restricts HIV-1 replication in macrophages by inhibiting dNTP biosynthesis pathway. Proc Natl Acad Sci USA. 2013; 110: E3997–4006. 10.1073/pnas.1306719110 24082141 10.1073/pnas.1306719110 PMC3801060

[41] Mashiba M, Collins DR, Terry VH, Collins KL. Vpr overcomes macrophage-specific restriction of HIV-1 Env expression and virion production. Cell Host Microbe. 2014; 16: 722–735. 10.1016/j.chom.2014.10.014 25464830 10.1016/j.chom.2014.10.014 PMC4269377

[42] Bouazzaoui A., Kreutz M., Eisert V., Dinauer N., Heinzelmann A., Hallenberger S, et al Stimulated trans-acting factor of 50 kDa (Staf50) inhibits HIV-1 replication in human monocyte-derived macrophages. Virology. 2006; 356: 79–94. 10.1016/j.virol.2006.07.025 16926043 10.1016/j.virol.2006.07.025

[43] Hayes MM, Lane BR, King SR, Markovitz DM, Coffey MJ. Peroxisome proliferator-activated receptor gamma agonists inhibit HIV-1 replication in macrophages by transcriptional and post- transcriptional effects. J Biol Chem. 2002; 277: 16913–16919. 10.1074/jbc.M200875200 11847231 10.1074/jbc.M200875200

[44] Skolnik PR, Rabbi MF, Mathys JM, Greenberg AS. Stimulation of peroxisome proliferator- activated receptors alpha and gamma blocks HIV-1 replication and TNFalpha production in acutely infected primary blood cells, chronically infected U1 cells, and alveolar macrophages from HIV-infected subjects. J Acquir Immune Defic Syndr. 2002; 31: 1–10. 12352144 10.1097/00126334-200209010-00001

[45] Alfano M, Sidenius N, Panzeri B, Blasi F, Poli G. Urokinase-urokinase receptor interaction mediates an ihnibitory signal for HIV-1 replication. Proc Natl Acad Sci USA. 2002; 99: 8862–8867. 10.1073/pnas.142078099 12084931 10.1073/pnas.142078099 PMC124389

[46] Nasr N., Maddocks S., Turville S.G., Harman A.N., Woolger N., Helbig K.J., et al HIV-1 infection of human macrophages directly induces viperin which inhibits viral production. Blood. 2012; 120: 778–788. 10.1182/blood-2012-01-407395 22677126 10.1182/blood-2012-01-407395

[47] Honda Y., Rogers L., Nakata K., Zhao B.Y., Pine R., Nakai Y., et al Type I interferon induces inhibitory 16-kD CCAAT/enhancer binding protein (C/EBP)beta, repressing the HIV-1 long terminal repeat in macrophages: pulmonary tuberculosis alters C/EBP expression, enhancing HIV-1 replicarion. J Exp Med. 1998; 188: 1255–1265. 9763605 10.1084/jem.188.7.1255 PMC2212491

[48] Sung TL, Rice AP. miR-198 inhibits HIV-1 gene expression and replication in monocytes and its mechanism of action appears to involve repression of cyclin T1. PLoS Pathog. 2009; 5: e1000263 10.1371/journal.ppat.1000263 19148268 10.1371/journal.ppat.1000263 PMC2607557

[49] Tada T, Zhang Y, Koyama T, Tobiume M, Tsunetsugu-Yokota Y., Yamaoka S, et al MARCH8 inhibits HIV-1 infection by reducing virion incorporation of envelope glycoproteins. Nat Med. 2015; 21: 1502–1507. 10.1038/nm.3956 26523972 10.1038/nm.3956

[50] Sukegawa S, Miyagi E, Bouamr F, Farkasová H, Strebel K. Mannose receptor 1 restricts HIV particle release from infected macrophages. Cell Rep. 2018; 22: 786–795. 10.1016/j.celrep.2017.12.085 29346774 10.1016/j.celrep.2017.12.085 PMC5792083

[51] Itzhaki R., Lin WR, Shang D, Wilcock GK, Faragher B, Jamieson GA, et al Herpes simplex virus type 1 in brain and risk of Alzheimer’s disease. Lancet. 1997; 349: 241–244. 10.1016/S0140-6736(96)10149-5 9014911 10.1016/S0140-6736(96)10149-5

[52] Burgos JS, Ramirez C, Sastre I, Valdivieso F. Effect of apolipoprotein E on the cerebral load of latent herpes simplex virus type 1 DNA. J Virol. 2006; 80: 5383–5387. 10.1128/JVI.00006-06 16699018 10.1128/JVI.00006-06 PMC1472141

[53] Corder EH, Robertson K, Lannfelt L, Bogdanovic N, Eggertsen G, Willkins J, et al HIV- infected subjects with the E4 allelle for APOE have excess dementia and peripheral neuropathy. Nat Med. 1998; 4: 1182–1184. 10.1038/2677 9771753 10.1038/2677

[54] Lin W.R., Wozniak M.A., Esiri M.M., Klenerman P., and Itzhaki R.F. Herpes simplex encephalitis: involvement of apolipoprotein E genotype. J Neuronal Neurosurg Psychiatry. 2001; 70: 117–119. 10.1136/jnnp.70.1.117 PMC1763452 11118260

[55] Burt TD, Agan BK, Marconi VC, He W, Kulkarni H, Mold JE, et al Apolipoprotein (apo) E4 enhances HIV-1 cell entry in vitro, and the APOE ε4/ε4 genotype accelerates HIV disease progression. Proc Natl Acad Sci USA. 2008; 105: 8718–8723. 10.1073/pnas.0803526105 18562290 10.1073/pnas.0803526105 PMC2438419

[56] Wozniak MA, Itzhaki RF, Faragher EB, James MW, Ryder SD, Irving WL, et al Apolipoprotein E-e4 protects against severe liver disease caused by hepatitis C virus. Hepatology. 2002; 36: 456–463. 10.1053/jhep.2002.34745 12143056 10.1053/jhep.2002.34745

[57] Chang KS, Jiang J, Cai Z, Luo G. Human apolipoprotein e is required for infectivity and production of hepatitis C virus in cell culture. J Virol. 2007; 81: 13783–13793. 10.1128/JVI.01091-07 17913825 10.1128/JVI.01091-07 PMC2168882

[58] Hishiki T, Shimizu Y, Tobita R, Sugiyama K, Ogawa K, Funami K, et al Infectivity of hepatitis C virus is influenced by association with apolipoprotein E isoforms. J Virol. 2010; 84: 12048–12057. 10.1128/JVI.01063-10 20826689 10.1128/JVI.01063-10 PMC2977863

[59] Zhang L, Yesupriya A, Chang MH, Teshale E, Teo CG. Apolipoprotein E and protection against hepatitis E viral infection in American non-Hispanic blacks. Hepatology. 2015; 62: 1346–1352. 10.1002/hep.27938 26096528 10.1002/hep.27938 PMC6686672

[60] Wozniak MA, Shipley SJ, Dobson CB, Parker SP, Scott FT, Leedham-Green M, et al Does apolipoprotein E determine outcome of infection by varicella zoster virus and by Epstein Barr virus. Eur J Hum Genet. 2007; 15: 672–678. 10.1038/sj.ejhg.5201812 17356546 10.1038/sj.ejhg.5201812

[61] Wozniak MA, Faragher EB, Todd JA, Koram KA, Riley EM, Itzhaki RF. Does apolipoprotein E polymorphism influence susceptibility to malaria? J Med Genet. 2003; 40: 348–351. 10.1136/jmg.40.5.348 12746397 10.1136/jmg.40.5.348 PMC1735474

[62] Roselaar SE, Daugherty A. Apolopoprotein E-deficient mice have impaired innate immune response to Listeria monocytogenes in vivo. J Lipid Res. 1998; 39: 1740–1743. 9741685

[63] de Bont N, Netea MG, Demacker PN, Verschueren I, Kullberg BJ, van Dijk KW, et al Apolipoprotein E knock-out mice are highly susceptible to endotoxemia and *Klebsiella pneumoniae* infection. J Lipid Res. 1999; 40: 680–685. 10191292

[64] Dobson CB, Sales SD, Hoggard P, Wozniak MA, Crutcher KA. The receptor-binding region of human apolipoprotein E has direct anti-infective activity. J Infect Dis. 2006; 193: 442–450. 10.1086/499280 16388493 10.1086/499280

[65] Kelly BA, Neil SJ, McKnight A, Santos JM, Sinnis P, Jack ER, et al Apolipoprotein E-derived antimicrobial peptide analogues with altered membrane affinity and increased potency and breadth of activity. FEBS J. 2007; 274: 4511–4525. 10.1111/j.1742-4658.2007.05981.x 17681018 10.1111/j.1742-4658.2007.05981.x

[66] Chertova E, Chertov O, Coren LV, Roser JD, Trubey CM, Bess JW Jr, et al Proteomic and biochemical analysis of purified human immunodeficiency virus type 1 produced from infected monocyte-derived macrophages. J Virol. 2006; 80: 9039–9052. 10.1128/JVI.01013-06 16940516 10.1128/JVI.01013-06 PMC1563931

[67] Koyanagi Y, O’Brien WA, Zhao JQ, Golde DW, Gasson JC, Chen IS, et al Cytokines alter production of HIV-1 from primary mononuclear phagocytes. Science. 1988; 241: 1673–1675. 3047875 10.1126/science.241.4873.1673

[68] Knott TJ, Rall SC, Innerarity TL, Jacobson SF, Urdea MS, Levy-Wilson B, et al Human apolopoprotein B: structure of carboxyl-terminal domains, sites of gene expression, and chromosomal localization. Science. 1985; 230: 37–43. 2994225 10.1126/science.2994225

[69] Sodroski J, Chun W, Rosen C, Campbell K, Haseltine WA. Role of the HTLV-III/LAV envelope in syncytium formation and cytopathicity. Nature. 1986; 322: 470–474. 10.1038/322470a0 3016552 10.1038/322470a0

[70] Schubert U, Bour S, Willey RL, Strebel K. Regulation of virus release by the macrophage- tropic human immunodeficiency virus type 1 AD8 isolate is redundant and can be controlled by either Vpu or Env. J Virol. 1999; 73: 887–896. 9882289 10.1128/jvi.73.2.887-896.1999 PMC103908

[71] Adachi A, Gendelman HE, Koenig S, Folks T, Willey R, Rabson A, et al Production of acquired immunodeficiency syndrome-associated retrovirus in human and nonhuman cells transfected with an infectious molecular clone. J Virol. 1986; 59: 284–291. 3016298 10.1128/jvi.59.2.284-291.1986 PMC253077

[72] von Schwedler U, Song J, Aiken C, Trono D. Vif is crucial for human immunodeficiency virus type 1 proviral DNA synthesis in infected cells. J Virol. 1993; 67: 4945–4955. 8331734 10.1128/jvi.67.8.4945-4955.1993 PMC237882

[73] Naldini L, Blömer U, Gallay P, Ory D, Mulligan R, Gage FH, et al In vivo gene delivery and stable transduction of nondividing cells by a lentiviral vector. Science. 1996; 272: 263–267. 8602510 10.1126/science.272.5259.263

[74] Chen CY, Shingai M, Welbourn S, Martin MA, Borrego P, Taveira N, et al Antagonism of BST-2/Tetherin is a conserved function of the Env glycoprotein of primary HIV-2 isolates. J. Virol. 2016; 90: 11062–11074. 10.1128/JVI.01451-16 27681141 10.1128/JVI.01451-16 PMC5126365

[75] Rowell JF, Stanhope PE, Siliciano RF. Endocytosis of endogenously synthesized HIV-1 envelope protein. Mechanism and role in processing for association with class II MHC. J Immunol. 1995; 155: 473–488. 7602119

[76] Eda Y, Murakami T, Nakasone T, Takizawa M, Someya K, Kaizu M, et al Anti-V3 humanized antibody KD-247 efficiently suppresses ex vivo generation of human immunodeficiency virus type 1 and affords sterile protection of monkeys against a heterogous simian/human immunodeficiency virus infection. J. Virol. 2006; 80: 5563–5570. 10.1128/JVI.02095-05 16699037 10.1128/JVI.02095-05 PMC1472178

[77] Hallenberger S, Bosch V, Angliker H, Shaw E, Klenk HD, Garten W. Inhibition of furin- mediated cleavage activation of HIV-1 glycoprotein gp160. Nature. 1992; 360: 358–361. 10.1038/360358a0 1360148 10.1038/360358a0

[78] Shih SJ, Allan C, Grehan S, Tse E, Moran C, Taylor JM. Duplicated downstream enhancers control expression of the human apolipoprotein E gene in macrophages and adipose tissue. J Biol Chem. 2000; 275: 31567–31572. 10.1074/jbc.M005468200 10893248 10.1074/jbc.M005468200

[79] Trusca VG, Fuior EV, Florea IC, Kardassis D, Simionescu M, Gafencu AV. Macrophage- specific up-regulation of apolipoprotein E gene expression by STAT1 is achieved via long range genomic interactions. J Biol Chem. 2011; 286: 13891–13904. 10.1074/jbc.M110.179572 21372127 10.1074/jbc.M110.179572 PMC3077590

[80] Trusca VG, Fuior EV, Fenyo IM, Kardassis D, Simionescu M, Gafencu AV. Differential action of glucocorticoids on apolipoprotein E gene expression in macrophages and hepatocytes. PLoS One. 2017; 12: e0174078 10.1371/journal.pone.0174078 28355284 10.1371/journal.pone.0174078 PMC5371326

[81] Gafencu A, Robciuc MR, Fuior E, Zannis VI, Kardassis D, Simionescu M. Inflammatory signaling pathways regulating apoE gene expression in macrophages. J Biol Chem. 2007; 282: 21776–21785. 10.1074/jbc.M611422200 17553793 10.1074/jbc.M611422200

[82] Singh NN, Ramji DP. Transforming growth factor-ß-induced expression of the apolipoprotein E gene requires c-Jun N-terminal kinase, p38 kinase, and casein kinase 2. Arterioscler Thromb Vasc Biol. 2006; 26: 1323–1329. 10.1161/01.ATV.0000220383.19192.55 16601234 10.1161/01.ATV.0000220383.19192.55

[83] Laffitte BA, Repa JJ, Joseph SB, Wilpitz DC, Kast HR, Mangelsdorf DJ, et al LXRs control lipid-inducible expression of the apolipoprotein E gene in macrophages and adipocytes. Proc Natl Acad Sci USA. 2001; 98: 507–512. 10.1073/pnas.98.2.507 11149950 10.1073/pnas.021488798 PMC14617

[84] Galetto R, Albajar M, Polanco JI, Zakin MM, Rodríguez-rey JC. Identification of a peroxisome-proliferator-activated-receptor response element in the apolipoprotein E gene control region. Biochem J. 2001; 357: 521–527. 11439103 10.1042/0264-6021:3570521 PMC1221980

[85] von Eckardstein A, Langer C, Engel T, Schaukal I, Cignarella A, Reinhardt J, et al ATP binding cassette transporter ABCA1 modulates the secretion of apolipoprotein E from monocyte-derived macrophages. FASEB J. 2011; 15: 1555–1561. 10.1096/fj.00-0798com 11427487

[86] Owens BJ, Anantharamaiah GM, Kahlon JB, Srinivas RV, Compans RW, Segrest JP. Apolipoprotein A-I and its amphipathic helix peptide analogues inhibit human immunodeficiency virus-induced syncytium formation. J Clin Invest. 1990; 86: 1142–1150. 10.1172/JCI114819 2170446 10.1172/JCI114819 PMC296843

[87] Martin I, Dubois MC, Saemark T, Ruysschaert JM. Apolipoprotein A-1 intercats with the N- terminal fusogenic domains of SIV (simian immunodeficiency virus) GP32 and HIV (human immunodeficiency virus) GP41: implications in viral entry. Biochem Biophys Res Commun. 1992; 186: 95–101. 1632797 10.1016/s0006-291x(05)80780-6

[88] Rose H, Hoy J, Woolley I, Tchoua U, Bukrinsky M, Dart A, et al HIV infection and high density lipoprotein metabolism. Atherosclerosis. 2008; 199: 79–86. 10.1016/j.atherosclerosis.2007.10.018 18054941 10.1016/j.atherosclerosis.2007.10.018 PMC2518204

[89] Bonnet E, Ruidavets JB, Tuech J, Ferriéres J, Collet X, Fauvel J, et al Apoprotein c-III and E-containing lipoparticles are markedly increased in HIV-infected patients treated with protease inhibitors: association with the development of lipodystrophy. J Clin Endocrinol Metab. 2001; 86: 296–302. 10.1210/jcem.86.1.7164 11232015 10.1210/jcem.86.1.7164

[90] Dong LM, Weisgraber KH. Human apolipoprotein E4 domain interaction. J Biol Chem. 1996; 271: 19053–19057. 8702576 10.1074/jbc.271.32.19053

[91] Finkelshtein D, Werman A, Novick D, Barak S, Rubinstein M. LDL receptor and its family members serve as the cellular receptors for vesicular stomatitis virus. Proc Natl Acad Sci USA. 2013; 110: 7306–7311. 10.1073/pnas.1214441110 23589850 10.1073/pnas.1214441110 PMC3645523

[92] Mahley RW, Ji ZS. Remnant lipoprotein metabolism: key pathways involving cell-surface heparan sulfate proteoglycans and apolipoprotein E. J Lipid Res. 1999; 40: 1–16. 9869645

[93] Patel M, Yanagishita M, Roderiquez G, Bou-Habib DC, Oravecz T, Hascall VC, et al Cell- surface heparan sulfate proteoglycan mediates HIV-1 infection of T-cell lines. AIDS Res Hum Retroviruses. 1993; 9: 167–174. 10.1089/aid.1993.9.167 8096145 10.1089/aid.1993.9.167

[94] Saphire AC, Bobardt MD, Zhang Z, David G, Gallay PA. Syndecans serve as attachment receptors for human immunodeficiency virus type 1 on macrophages. J Virol. 2001; 75: 9187–9200. 10.1128/JVI.75.19.9187-9200.2001 11533182 10.1128/JVI.75.19.9187-9200.2001 PMC114487

[95] Jiang J, Cun W, Wu X, Shi Q, Tang H, Luo G, et al Hepatitis C virus attachment mediated by apolipoprotein E binding to cell surface heparan sulfate. J Virol. 2012; 86: 7256–7267. 10.1128/JVI.07222-11 22532692 10.1128/JVI.07222-11 PMC3416335

[96] Rosa A, Chande A, Ziglio S, De Sanctis V, Bertorelli R, Goh SL, et al HIV-1 Nef promotes infection by excluding SERINC5 from virion incorporation. Nature. 2015; 526: 212–217. 10.1038/nature15399 26416734 10.1038/nature15399 PMC4861059

[97] Usami Y, Wu Y, Göttlinger HG. SERINC3 and SERINC5 restrict HIV-1 infectivity are contercated by Nef. Nature. 2015; 526: 218–223. 10.1038/nature15400 26416733 10.1038/nature15400 PMC4600458

[98] van den Elzen P, Garg S, León L, Brigl M, Leadbetter EA, Gumperz JE, et al Apolipoprotein-mediated pathways of lipid antigen presentation. Nature. 2005; 437: 906–910. 10.1038/nature04001 16208376 10.1038/nature04001

[99] van Niel G, Bergam P, Di Cicco A, Hurbain I, Lo Cicero A, Dingli F, et al Apolipoprotein E regulates amyloid formation within endosomes of pigment cells. Cell Rep. 2015; 13: 43–51. 10.1016/j.celrep.2015.08.057 26387950 10.1016/j.celrep.2015.08.057

[100] Lee CY, Tse W, Smith JD, Landreth GE. Apolipoprotein E promotes beta-amyloid trafficking and degradation by modulating microglial cholesterol levels. J Biol Chem. 2012; 287: 2032–2044. 10.1074/jbc.M111.295451 22130662 10.1074/jbc.M111.295451 PMC3265883

[101] Tedbury PR, Freed EO. The role of matrix in HIV-1 envelope glycoprotein incorporation.Trends Microbiol. 2014; 22: 372–378. 10.1016/j.tim.2014.04.012 24933691 10.1016/j.tim.2014.04.012 PMC4157688

[102] Hashimoto M, Bhuyan F, Hiyoshi M, Noyori O, Nasser H, Miyazaki M, et al Potential role of the formation of tunneling nanotubes in HIV-1 spread in macrophages. J Immunol. 2016; 196: 1832–1841. 10.4049/jimmunol.1500845 26773158 10.4049/jimmunol.1500845

[103] Hashimoto M, Nasser H, Bhuyan F, Kuse N, Satou Y, Harada S, et al Fibrocytes differ from macrophages but can be infected with HIV-1. J Immunol. 2015; 195: 4341–4350. 10.4049/jimmunol.1500955 26416279 10.4049/jimmunol.1500955

[104] Ariumi Y, Masutani M, Copeland TD, Mimori T, Sugimura T, Shimotohno K, et al Suppression of the poly(ADP-ribose) polymerase activity by DNA-dependent protein kinase in vitro. Oncogene. 1999; 18: 4616–4625. 10.1038/sj.onc.1202823 10467406 10.1038/sj.onc.1202823

[105] Ariumi Y, Kaida A, Lin JY, Hirota M, Masui O, Yamaoka S, Taya Y, Shimotohno K. HTLV- 1 Tax oncoprotein represses the p53-mediated trans-activation function through coactivator CBP sequestration. Oncogene. 2000; 19: 1491–1499. 10.1038/sj.onc.1203450 10734308 10.1038/sj.onc.1203450

[106] Ariumi Y, Kuroki M, Abe K, Dansako H, Ikeda M, Wakita T, et al DDX3 DEAD-box RNA helicase is required for hepatitis C virus RNA replication. J Virol. 2007; 81: 13922–13926. 10.1128/JVI.01517-07 17855521 10.1128/JVI.01517-07 PMC2168844

[107] Ariumi Y, Kuroki M, Kushima Y, Osugi K, Hijikata M, Maki M, et al Hepatitis C virus hijaks P-body and stress granule components around lipid droplets. J. Virol. 2011; 85: 6882–6892. 10.1128/JVI.02418-10 21543503 10.1128/JVI.02418-10 PMC3126564

[108] Alam M, Kuwata T, Shimura K, Yokoyama M, Ramirez Valdez KP, Tanaka K, et al Enhanced antibody-mediated neutralization of HIV-1 variants that are resistant to fusion inhibitors. Retrovirology. 2016; 13: 70 10.1186/s12977-016-0304-7 27670680 10.1186/s12977-016-0304-7 PMC5037607

[109] Zufferey R, Nagy D, Mandel RJ, Naldini L, Trono D. Multiply attenuated lentiviral vector achieves efficient gene delivery in vivo. Nat Biotechnol. 1997; 15: 871–875. 10.1038/nbt0997-871 9306402 10.1038/nbt0997-871

[110] Ariumi Y, Turelli P, Masutani M, Trono D. DNA damage sensors ATM, ATR, DNA-PKcs, and PARP-1 are dispensable for human immunodeficiency virus type 1 integration. J. Virol. 2005; 79: 2973–2978. 10.1128/JVI.79.5.2973-2978.2005 15709017 10.1128/JVI.79.5.2973-2978.2005 PMC548471

[111] Ariumi Y, Kuroki M, Dandako H, Abe K, Ikeda M, Wakita T, Kato N. The DNA damage sensors ataxia-telangiectasia mutated kinase and checkpoint kinase 2 are required for hepatitis C virus RNA replication. J. Virol. 2008; 82: 9639–9646. 10.1128/JVI.00351-08 18667510 10.1128/JVI.00351-08 PMC2546985

